# The *Drosophila melanogaster* Gut Microbiota Provisions Thiamine to Its Host

**DOI:** 10.1128/mBio.00155-18

**Published:** 2018-03-06

**Authors:** David R. Sannino, Adam J. Dobson, Katie Edwards, Esther R. Angert, Nicolas Buchon

**Affiliations:** a Department of Microbiology, Cornell University, Ithaca, New York, USA; b Institute of Healthy Ageing, University College London, London, United Kingdom; c Department of Entomology, Cornell University, Ithaca, New York, USA; d Cornell Institute of Host-Microbe Interactions and Disease, Ithaca, New York, USA; University of Hawaii at Manoa

**Keywords:** *Acetobacter*, *Drosophila*, gut microbiota, synthetic diet, thiamine, vitamin B_1_

## Abstract

The microbiota of *Drosophila melanogaster* has a substantial impact on host physiology and nutrition. Some effects may involve vitamin provisioning, but the relationships between microbe-derived vitamins, diet, and host health remain to be established systematically. We explored the contribution of microbiota in supplying sufficient dietary thiamine (vitamin B_1_) to support *D. melanogaster* at different stages of its life cycle. Using chemically defined diets with different levels of available thiamine, we found that the interaction of thiamine concentration and microbiota did not affect the longevity of adult *D. melanogaster*. Likewise, this interplay did not have an impact on egg production. However, we determined that thiamine availability has a large impact on offspring development, as axenic offspring were unable to develop on a thiamine-free diet. Offspring survived on the diet only when the microbiota was present or added back, demonstrating that the microbiota was able to provide enough thiamine to support host development. Through gnotobiotic studies, we determined that *Acetobacter pomorum*, a common member of the microbiota, was able to rescue development of larvae raised on the no-thiamine diet. Further, it was the only microbiota member that produced measurable amounts of thiamine when grown on the thiamine-free fly medium. Its close relative *Acetobacter pasteurianus* also rescued larvae; however, a thiamine auxotrophic mutant strain was unable to support larval growth and development. The results demonstrate that the *D. melanogaster* microbiota functions to provision thiamine to its host in a low-thiamine environment.

## IMPORTANCE

There has been a long-standing assumption that the microbiota of animals provides their hosts with essential B vitamins; however, there is not a wealth of empirical evidence supporting this idea, especially for vitamin B_1_ (thiamine). To determine whether this assumption is true, we used *Drosophila melanogaster* and chemically defined diets with different thiamine concentrations as a model. We found that the microbiota does provide thiamine to its host, enough to allow the development of flies on a thiamine-free diet. The power of the *Drosophila*-microbiota system allowed us to determine that one microbiota member in particular, *Acetobacter pomorum*, is responsible for the thiamine provisioning. Thereby, our study verifies this long-standing hypothesis. Finally, the methods used in this work are applicable for interrogating the underpinnings of other aspects of the tripartite interaction between diet, host, and microbiota.

## INTRODUCTION

The interplay between animals and microbes has helped forge the evolution of metazoans (1). Throughout their life, animals are in constant contact with microorganisms, and these interactions are dynamic. The microbiota influences immune, developmental, and metabolic functions of the host through a plethora of secondary metabolites and molecules; however, it often remains unclear what secondary metabolites and what metabolic functions are affected by which member of the microbiota.

Recently, *Drosophila melanogaster* has emerged as a potent model to study the function of gut microbes (2). The gut microbiota of *D. melanogaster* reared in the laboratory is variable (3, 4), but this low-diversity community is generally dominated by a few members of the *Lactobacillaceae* and *Acetobacteraceae* (3, 5–9). The microbiota impacts host physiology and development as it primes the immune system (10), shapes gut morphology and homeostasis (3, 10, 11), and even influences mating preferences (12). In *D. melanogaster*, the nutritional impacts of its microbiota are substantial (9, 13, 14). Through manipulation of host signaling pathways, *Acetobacter* spp. can accelerate host development, increase body size and growth rate, and help regulate host glucose and lipid levels (6, 9, 15, 16). *Acetobacter pomorum* possibly achieves this through the production of acetic acid, which influences the nutrient-sensing insulin/insulin-like growth factor signaling pathway (6). When *D. melanogaster* is raised in nutrient-poor conditions, *Lactobacillus plantarum* modulates the activity of the target of rapamycin (TOR) signaling pathway, enhancing the production of hormonal signals that hasten larval development and growth rate (7). Microbiota composition also influences host nutritional phenotypes (17). For instance, *Acetobacter* spp. maintain triacylglycerol (TAG) levels inside the host in a dose-dependent manner, and this metabolic response is stimulated by cocolonization of the gut with *Lactobacillus* species (16, 17). Microbiota-driven dietary modification, particularly the reduction of dietary glucose by microbial oxidation, also accounts for metabolic responses of the host (18, 19).

Despite the positive impacts the microbiota has on *D. melanogaster* nutrition and physiology, there are tradeoffs imposed by this close association. In the gut, *Drosophila* produces reactive oxygen species (20) and antimicrobial peptides (AMPs) (21) to combat infection and control gut microbiota (10); however, the stress induced by the presence of microbes damages the epithelium, leading to increased epithelial cell turnover (11). As flies age, their bacterial load increases (3, 8, 20, 22), contributing to a shift from epithelium renewal to dysplasia, which eventually compromises gut integrity (11, 20, 22, 23). The onset of the loss of barrier function coincides with an increase in *Gammaproteobacteria* at the expense of *Firmicutes* (22). Barrier dysfunction can in turn lead to increased *Alphaproteobacteria* (*Acetobacter*) abundance, a concomitant rise in the immune response, and ultimately host death (20, 22). Aging also results in a loss of immune regulation, with the persistent activation of the immune deficiency (Imd) pathway, resulting in chronic expression of AMPs (11, 20). In contrast, axenic flies have a depressed immune response (11, 20) and a longer life span than their conventionally raised counterparts (22). Old axenic flies exhibit healthier, less deteriorated guts, as there is reduced epithelial dysplasia, and intestinal stem cell proliferation rates remain similar to the rates seen in younger flies (11, 20).

It has long been hypothesized that the gut microbiota of animals provides B vitamins to their hosts (24). Recent studies have implicated the *Drosophila* microbiota in supplying folate (14, 25) and riboflavin (9, 13); however, the contribution of thiamine (vitamin B_1_) from the microbiota is not well understood. Thiamine is necessary for cellular life, as its diphosphorylated form, thiamine pyrophosphate (TPP), serves as a cofactor for enzymes involved in essential cellular processes, including energy metabolism and the biosynthesis of the precursors for nucleotides, amino acids, and other cellular compounds (26–28). Consequently, thiamine deficiency results in disease and death in animals, including humans (28–31). Many bacteria, archaea, fungi, and plants, can synthesize thiamine (32), while animals typically acquire thiamine through their diet. In ruminants, however, the majority of the thiamine entering the duodenum originates from the rumen microbiota (33–35); this thiamine is released when rumen microbes are lysed in the abomasum, making it available for absorption in the small intestine. In humans, dietary thiamine is taken up by transporters in small intestine enterocytes (36) and in the colon where colonocytes have an uptake system specific for TPP (26). Genomic evidence indicates that colonic bacteria are enriched in enzymes for thiamine synthesis, suggesting that the gut microbiota may be a thiamine source for the host (37, 38). However, a comprehensive understanding of how the microbiota impacts thiamine metabolism in animal systems is still lacking.

*Drosophila melanogaster* provides a rigorous genetic and physiological system for interrogating the relationship between an animal host, its microbiota, and diet. The low-diversity microbial community of *D*. *melanogaster* and the ease of generating axenic animals (39) and reconstituting microbiota in axenic flies allow for the functional characterization and identification of dietary contributions of individual members of the microbiota (3, 16, 17). With the advent of a robust chemically defined diet (25, 40), it is now possible to fully examine how each dietary component contributes to this intricate tripartite interaction (40).

In this study, we used a chemically defined diet with conventionally raised (CR) and axenic (Ax) flies to determine the impact of dietary thiamine and microbiota on the development, longevity, and egg production of adult *D. melanogaster*. We found that Ax flies had a longer life span than their CR counterparts, but this was not thiamine dependent. There was also no discernible dietary thiamine by microbiota effect on female egg production. The larval period greatly impacts adult fitness, as larval stores are carried into adulthood (41), so we investigated the impact of dietary thiamine and the microbiota on development of the progeny from eggs. Axenic larvae developing on the thiamine-free diet were unable to pupate; however, CR larvae were able to pupate and develop into adults. This demonstrated that the microbiota provisioned thiamine to its host, allowing for larval development and growth into adulthood. To determine which members of the microbiota provided thiamine, we generated monoassociated flies and monitored development on the thiamine-free diet. We found that development was rescued only when eggs were inoculated with *A. pomorum*, either in monoassociation or polyassociation, and that *A. pomorum* was the only microbiota member that produced thiamine on the chemically defined diet. Additional monoassociation studies with wild-type *Acetobacter pasteurianus*, a thiamine auxotrophic strain, confirmed that developmental rescue was due to thiamine provisioning by *Acetobacter* spp. These results establish that the *D. melanogaster* microbiota functions as a source of thiamine for its host in a low-thiamine environment.

## RESULTS

### The interaction of thiamine and microbiota does not significantly affect adult life span.

**FIG 1:**
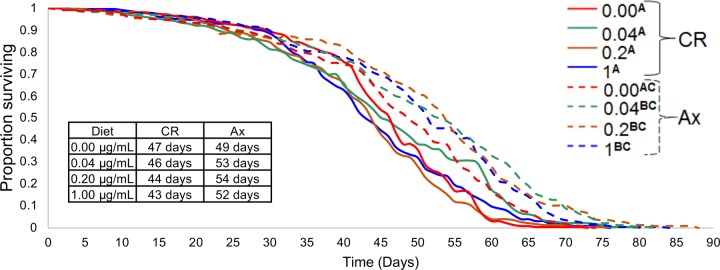
Impact of dietary thiamine on the longevity of conventionally reared (CR) and axenic (Ax) flies. Survival curves of CR and Ax flies on four diets with different vitamin B_1_ concentrations (0.00, 0.04, 0.2, and 1.0 µg/ml) indicate little difference between treatments. The 95% Wald confidence limits (CL) show significant differences when comparing CR and Ax flies to each other on the 0.04-, 0.2-, and 1.0-µg/ml thiamine diets. Thiamine treatments with the same superscript letters in the key are not significantly different; for example, treatments with the AC label are not significantly different from the treatments with the A or C superscript. The inset table shows the median survival time for the CR and Ax flies. Point estimates and CL are provided in Table S1 in the supplemental material.

In animals, thiamine is essential for energy metabolism and proper cell function (28, 42, 43). We thus hypothesized that thiamine deficiency would alter the life span of *Drosophila* but that microbial thiamine provision would rescue this effect. To determine the role of thiamine content in food and the impact of gut microbes on thiamine availability, axenic (Ax) and conventionally raised (CR) flies were fed one of four chemically defined diets with different concentrations of thiamine (0.00, 0.04, 0.2, or 1.0 µg/ml). Consistent with previous reports, Ax flies on all diets were longer-lived than CR flies (Fig. 1), which may be attributed to deleterious effects of the microbiota on gut barrier function (11, 20, 22). When comparing Ax and CR flies on each diet, we observed significantly longer life spans of the Ax flies on the 0.04-, 0.2-, and 1.0-µg/ml thiamine diets; however, there was no statistically significant difference between the life spans of the Ax and CR flies on the no-thiamine diet (Fig. 1). Further, there was no significant effect of the thiamine by microbiota interaction term. From this, we concluded that dietary thiamine does not influence the life span of adult *D. melanogaster*, only the presence or absence of the microbiota does.

10.1128/mBio.00155-18.6 TABLE S1  Point estimates and upper and lower confidence limits for the pairwise comparisons. Significant differences do not have a value of 1 within the confidence limits, and they are highlighted in yellow. Download TABLE S1, TIF file, 8.3 MB. Copyright © 2018 Sannino et al. 2018 Sannino et al. This content is distributed under the terms of the Creative Commons Attribution 4.0 International license.

### Dietary thiamine and microbiota do not impact female egg production.

**FIG 2:**
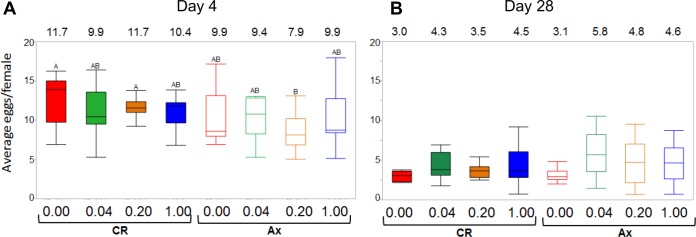
Impact of dietary thiamine and microbiota on egg production in adult flies. (A and B) The average number of eggs/female/tube was determined after 4 days (A) and 28 days (B) on the diets for both CR and Ax flies. Box plots depicting egg production are shown. The values at the top of the graphs are the average number of eggs/female for each treatment. Treatments designated with the same letter are not significantly different. After 4 days, axenic flies raised on 0.2 µg/ml thiamine produced significantly fewer eggs (*P* value < 0.0017) compared to CR flies on the same diet (*P* = 0.009) and the CR flies on the no-thiamine diet (*P* = 0.0013). There were no significant differences observed in egg production for any treatments after 28 days.

In insects, including *D. melanogaster*, egg production is an energetically intensive process impacted by female metabolic status (44, 45) and promoted by dietary B vitamins (25, 46). Therefore, we hypothesized that egg production (measured by egg laying) could be dependent on the supply of dietary thiamine and may be enhanced by the presence of the microbiota. Specifically, we predicted that Ax flies would lay fewer eggs than CR flies, and egg output would be disproportionately impacted by reduction of dietary thiamine in Ax flies relative to CR flies. To test this, we counted eggs laid by flies from the longevity study and analyzed egg production via an analysis of variance (ANOVA) (Fig. 2). For each diet, the average numbers of eggs produced by 4-day-old females were lower for Ax flies than for CR flies (*P* < 0.01), but this was not thiamine dependent (Fig. 2A). These findings suggest that in early adulthood, there is no interaction between thiamine and microbiota affecting egg production.

It has been previously shown that starved *D. melanogaster* females are able to produce eggs into the sixth day of adulthood from nutrients stored from their larval period (47). This raises the possibility that we did not detect an effect of a lack of dietary thiamine on egg production due to thiamine stores in the fly. As flies age and exhaust their larval supplies, they could become more reliant on their diet for nutrition and egg production. On this basis, we hypothesized that older, axenic females would produce fewer eggs when dietary thiamine was lacking but that this defect could be alleviated by the presence of the microbiota. Therefore, we examined egg production of older females that have been kept on their respective diets for 4 weeks (Fig. 2B). The ANOVA indicated there was no statistically significant interaction of microbiota and dietary thiamine on egg laying. These results suggest that egg production is not a function of thiamine concentration and microbiota in older females. The data further intimate that thiamine has no effect on the age-related degeneration of egg production.

### Thiamine is essential for offspring development and survival.

**FIG 3:**
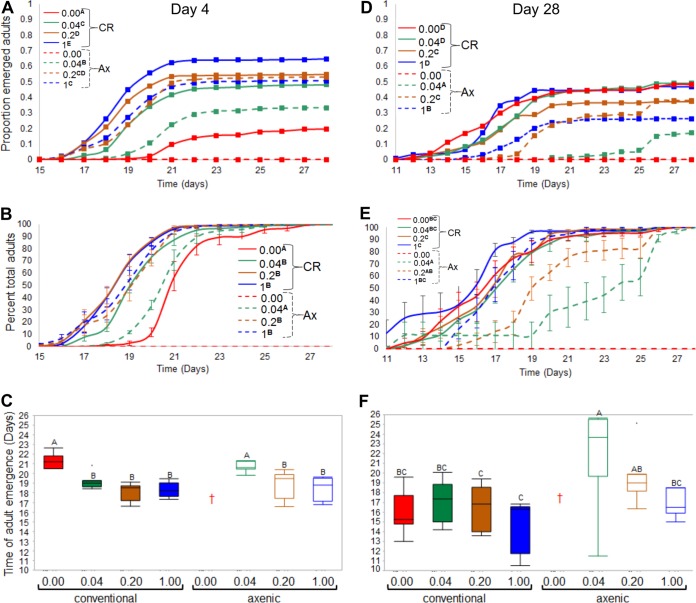
Impact of dietary thiamine and microbiota on fly development. Development to adulthood was assayed for CR and Ax eggs raised on diets containing different concentrations of thiamine. For all plots, treatments designated with the same letter are not significantly different from one another. (A and D) Survival curves depicting the proportion of emerged adults from eggs laid at 4 and 28 days, respectively. Overall, CR flies survived better than their Ax counterparts on any thiamine diet, with comparisons shown in Table S2 (eggs laid on day 4) and Table S3 (eggs laid on day 28). (B and E) Time course for adult emergence from eggs laid by 4- and 28-day-old females, respectively. Each value shows the mean ± standard error of the mean (SEM) (error bar) of the proportion of the total adults that emerged in a given treatment. (C and F) Box plots representing the time of emergence of 50% of the total population of adults from the eggs laid at 4 days and 28 days, respectively (*P* < 0.0023 by *t* test). The red dagger indicates that no flies survived to adulthood. In panel C, adult emergence of CR flies raised on the no-thiamine diet and Ax flies raised on the 0.04-µg/ml thiamine diet were significantly delayed compared to all fly trials that produced adults (*P* < 0.0001). (F) For eggs from older females, axenic flies on the 0.04-µg/ml thiamine diet emerged later than the CR flies on all other diets (*P* < 0.0001).

The number of eggs produced is only an indirect measure of fecundity, as it does not gauge offspring quality, or vigor. It is therefore possible that F1 progeny of thiamine-deprived flies are more susceptible to lack of thiamine in the diet. We hypothesized that Ax larvae raised in a low-thiamine environment would have decreased fitness, with the potential for the defect to be rescued by the transformation of the diet through the supply of thiamine by the microbiota. Hence, we examined how the microbiota and thiamine levels influence the development of eggs to adulthood on our four diets. In contrast to the egg-laying assays, this assay uncovered a substantial diet by microbiota interaction. Offspring from axenic eggs laid on the no-thiamine diet did not survive, as the larvae melanized and died prior to pupation (Fig. 3; also see Fig. S1 in the supplemental material). However, when the microbiota was present, eggs laid on this diet developed into pupae and adults. This pattern of microbiota rescue was observed both for eggs laid by flies that were on the diets for 4 days (Fig. 3A to C) and 28 days (Fig. 3D to F), suggesting that the microbiota transforms the diet through thiamine production. These results confirm that dietary thiamine is required for larval development in Ax flies (46) and further demonstrate that microbiota-derived thiamine is sufficient for development.

10.1128/mBio.00155-18.1 FIG S1  Larval growth of CR versus Ax flies on the no-thiamine diet. Eggs from CR flies were placed in the tube on the left, and eggs from Ax flies were placed in the tube on the right. As the CR larvae grew, they churned up the food as they ate. The food in the Ax tube is not as well churned as the food in the CR tube, the medium appears to be drying out, and dead melanized larvae are visible on the food surface. Download FIG S1, TIF file, 7.9 MB. Copyright © 2018 Sannino et al. 2018 Sannino et al. This content is distributed under the terms of the Creative Commons Attribution 4.0 International license.

10.1128/mBio.00155-18.7 TABLE S2  CoxPH comparisons of survival to adulthood of eggs laid on day 4. Statistically significant values are indicated as follows: ***, <0.001; **, <0.01; *, <0.05; ., <0.1. Values without asterisks or a period have a value of 1. Download TABLE S2, TIF file, 16.5 MB. Copyright © 2018 Sannino et al. 2018 Sannino et al. This content is distributed under the terms of the Creative Commons Attribution 4.0 International license.

10.1128/mBio.00155-18.8 TABLE S3  CoxPH comparisons of survival to adulthood of eggs laid on day 28. Statistically significant values are indicated as follows: ***, <0.001; **, <0.01; *, <0.05; ., <0.1. Values without asterisks or a period have a value of 1. Download TABLE S3, TIF file, 15.4 MB. Copyright © 2018 Sannino et al. 2018 Sannino et al. This content is distributed under the terms of the Creative Commons Attribution 4.0 International license.

Nutrients influence not only the developmental success of larvae but also their developmental rate (13). Consequently, we next hypothesized that the timing of eggs to pupation and adulthood could be a function of microbiota and dietary thiamine. When 0.4 μg/ml thiamine was present in the medium, CR flies developed faster than their Ax counterparts for eggs laid either at 4 days (Fig. 3B and C and Fig. S2B and C) or 4 weeks of age (Fig. 3E and F and Fig. S2E and F). This suggests that the microbiota enhances the development rate when dietary thiamine is low. For the eggs laid at 4 days, development was slower on the no-thiamine diet than any other condition, and the time to 50% pupation and adult emergence was significantly longer than all other conditions except for the Ax flies raised on the 0.04-µg/ml thiamine medium (Fig. 3B and C and Fig. S2B and C). We did not observe the same developmental trend with the eggs laid at 4 weeks, as all CR flies developed at similar rates (Fig. 3E and F and Fig. S2E and F). Strikingly, larvae from eggs laid at 4 weeks developed faster than larvae from eggs laid at 4 days. The differences in developmental rate imply that the microbiota plays a larger role in mitigating the dietary thiamine defect, as the development of eggs laid by older mothers is less constrained than the eggs laid by younger mothers. This may be a function of the greater bacterial load in older flies (3, 8, 20, 22). In contrast, Ax fly development is more contingent upon dietary thiamine, as the speed of development ranks with the concentration of thiamine in the medium for eggs laid at both times (Fig. 3B and E and Fig. S2B and E).

10.1128/mBio.00155-18.2 FIG S2  Impact of dietary thiamine and microbiota on fly development. Development to pupation was assayed for CR and Ax eggs raised on diets with different concentrations of thiamine. For all plots, treatments in keys designated with the same letter are not significantly different from one another. (A and D) Survival curves depicting the proportion of pupae from eggs laid at 4 and 28 days, respectively. Overall, CR flies survived better than their Ax counterparts on any thiamine diet, with comparisons in Table S4 (day 4 eggs) and Table S5 (day 28 eggs). (B and E) Time for 100% of progeny to reach pupation from eggs laid by females at 4 and 28 days, respectively. Each value shows the mean ± SEM (error bar) of the proportion of progeny to pupate in a given treatment. (C and F) Box plots representing the time of emergence of 50% of the total population of pupae from the eggs laid at 4 days and 28 days, respectively (*P* value of <0.0023 by *t* test). The red dagger indicates that no flies survived to adulthood. In panel C, the time of emergence on the 0.00-µg/ml thiamine diet was significantly slower than for all the other conventional diets and the axenic 0.2-µg/ml and 1-µg/ml thiamine diets with *P* < 0.0001 for each comparison. The time of emergence on the 0.04-µg/ml thiamine diet was significantly slower than all the other conventional diets than what was previously mentioned, and all the other axenic diets (*P* < 0.0001 for all comparisons). (F) The time of emergence on the 0.04-µg/ml thiamine diet was significantly slower than for all the other conventional and axenic diets with *P* < 0.0001 for all comparisons. The time of emergence on the axenic 0.2-µg/ml thiamine diet was slower than those on the conventional 1-µg/ml and 0.00-µg/ml thiamine diets (*P* < 0.0001 and *P* = 0.001, respectively), and the time of emergence on the axenic 1-µg/ml thiamine diet was slower than the time for its conventional counterpart (*P* = 0.0021). Download FIG S2, TIF file, 13.7 MB. Copyright © 2018 Sannino et al. 2018 Sannino et al. This content is distributed under the terms of the Creative Commons Attribution 4.0 International license.

10.1128/mBio.00155-18.9 TABLE S4  CoxPH comparisons of survival to pupae of eggs laid on day 4. Statistically significant values are indicated as follows: ***, <0.001; **, <0.01; *, <0.05; ., <0.1. Values without asterisks or a period have a value of 1. Download TABLE S4, TIF file, 16.5 MB. Copyright © 2018 Sannino et al. 2018 Sannino et al. This content is distributed under the terms of the Creative Commons Attribution 4.0 International license.

10.1128/mBio.00155-18.10 TABLE S5  CoxPH comparisons of survival to pupae of eggs laid on day 28. Statistically significant values are indicated as follows: ***, <0.001; **, <0.01; *, <0.05; ., <0.1. Values without asterisks or a period have a value of 1. Download TABLE S5, TIF file, 15.4 MB. Copyright © 2018 Sannino et al. 2018 Sannino et al. This content is distributed under the terms of the Creative Commons Attribution 4.0 International license.

10.1128/mBio.00155-18.3 FIG S3  Impact of microbiota reassociation on fly development on the no-thiamine diet. (A) Proportion of gnotobiotic eggs that pupated when reassociated with microbiota members. Significant differences from CoxPH modeling are depicted as letter superscripts next to the reassociations in the keys. The survival to pupation is significantly higher in the *A. pomorum*-monoassociated gnotobiotic flies (*P* < 0.001). (C) Proportion of gnotobiotic eggs that pupated when associated with wild-type and mutant *A. pasteurianus*. (B and D) Developmental progression as the time to pupation in the microbiota and *A. pasteurianus* associations, respectively. In panel B, no flies developed when associated with the mutant. Values are means ± SEM. Download FIG S3, TIF file, 9.8 MB. Copyright © 2018 Sannino et al. 2018 Sannino et al. This content is distributed under the terms of the Creative Commons Attribution 4.0 International license.

As with the speed of development, the microbiota alters the diet to promote the survivorship of eggs to pupation and adulthood. When comparing Ax and CR eggs laid at 4 days on each diet, we observed significantly more pupation and adult emergence of CR flies than Ax flies on almost every diet (Fig. 3A and Fig. S2A), demonstrating that the interaction of microbiota with diet elevates survival. The enhancement was a function of thiamine concentration, as more thiamine typically equated to higher survivorship in the day 4 eggs (Fig. 3A). For the Ax eggs laid at 4 days, thiamine was the major driver of survivorship. When observing development of the eggs laid at 28 days, there was a universal decrease in the total survivorship to pupation (Fig. S2A and S2D) and adulthood (Fig. 3A and D) for all diets except for the CR flies on the 0.00- and 0.04-µg/ml thiamine diets. On these diets, total survivorship to pupation and adulthood was increased and statistically equivalent to the 1-μg/ml thiamine diet. The lack of a difference in survivorship between the diets supports the idea that the presence of the microbiota has a greater influence on the eggs laid by older females, as this is not dependent on thiamine concentration but on the presence of the microbiota (Fig. 3D). However, unlike the development rate, survival of Ax eggs was not proportional to thiamine concentration, as the 0.2-µg/ml thiamine diet allowed for significantly more Ax adults to develop than the 1-µg/ml thiamine diet (Fig. 3D), suggesting that there may be an optimal dietary thiamine concentration to support development of Ax flies.

### *Acetobacter pomorum* rescues development of *D. melanogaster* on a thiamine-free diet.

**FIG 4:**
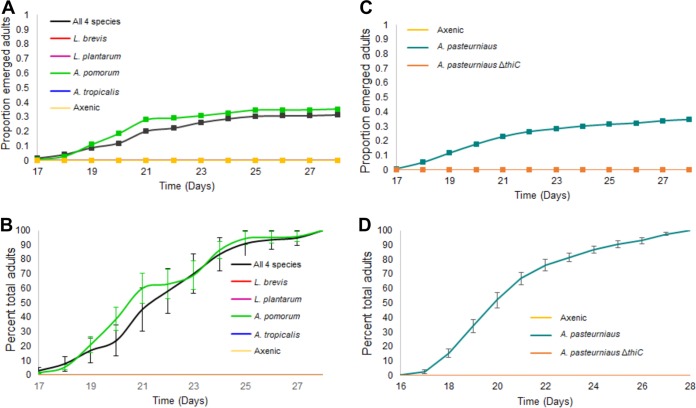
Impact of microbiota reassociation on fly development on the no-thiamine diet. (A) Proportion of gnotobiotic eggs that emerged as adults when reassociated with microbiota members. Significant differences from Cox proportional hazards (CoxPH) modeling are depicted as letter superscripts next to the reassociations in the keys. The survival to adulthood is significantly higher in the *A. pomorum*-monoassociated gnotobiotic flies (*P* < 0.001). (C) Proportion of gnotobiotic eggs that emerged as adults when associated with wild-type and mutant *A. pasteurianus*. (B and D) Developmental progression as the time to adult emergence in the microbiota and *A. pasteurianus* associations, respectively. No flies developed when associated with the mutant (B). Values are means ± SEM.

We next asked whether any one species in the laboratory microbiota is sufficient to rescue the developmental effects of thiamine deficiency. In our laboratory, the *D. melanogaster* microbiota is dominated by *A. pomorum*, *Acetobacter tropicalis*, *Lactobacillus brevis*, and *L. plantarum*. Both *Acetobacter* spp. have the genomic potential to produce thiamine, whereas the lactobacilli do not (15). Therefore, we hypothesized that either *Acetobacter* species would rescue development on a thiamine-free diet. To test this, we generated gnotobiotic flies by reassociating Ax eggs with each individual microbiota member or all four members of the microbiota on the no-thiamine diet. Only *A. pomorum* rescued development (Fig. 4). When the other bacteria were added in monoassociations, the larvae did not develop into pupae and died (Fig. S5C). Furthermore, *A. pomorum* alone was as effective at rescuing development as adding all four species together (Fig. 4). Importantly, the females that laid these eggs were reared and fed thiamine-replete media prior to oviposition, and yet their offspring were still subject to the effects of *A. pomorum*, suggesting that exogenous thiamine—whether it originates from the microbiota or diet—is more important for development than thiamine provisioned to the egg by the mother.

When dietary thiamine was present, development was not substantially affected by the presence of individual microbiota members or by having all four members present (Fig. S4B and C and Fig. S4E and F). No community member enhanced development; in contrast, the Ax treatment had significantly higher survival of pupae and adults (Fig. S4A and S4D). For instance, *L. brevis* had a negative effect on development, as gnotobiotic flies exhibited significantly reduced survival of adults compared to their Ax counterparts (Fig. S4D). Our results suggest that *L. brevis* may compete with its host for thiamine, limiting the amount available for host consumption.

10.1128/mBio.00155-18.4 FIG S4  Impact of microbiota on fly development on the 0.2-µg/ml thiamine diet. (A and D) Proportion of gnotobiotic eggs that have pupated (A) and proportion of eggs that emerged as adults (D). Significant differences from CoxPH modeling are displayed as letter superscripts next to the reassociations in the keys. In panel A, for all statistically significant pairwise comparisons of proportion pupated, *P* < 0.001, except for *L. plantarum* versus axenic (*P* = 0.00131). In panel D, for all statistically significant pairwise comparisons of proportion adult emergence, *P* < 0.001, except for *L. brevis* versus *A*. *pomorum* (*P* = 0.00206), *A. tropicalis* (*P* = 0.02282), all four species (*P* = 0.00328), and *L. plantarum* (*P* = 0.03303), and axenic versus *A. tropicalis* (*P* = 0.00168). (B and E) Development as the average time to pupation (B) and adult emergence (E). Each value shows the average proportion of eggs to pupate (B) or average proportion of adults to emerge (E). Values are means ± standard errors (*n* = 6 for each point). (C and F) Box plots representing the time of pupation (C) and adult emergence of 50% of the total population (F). The *A. pomorum* gnotobiotic pupae develop significantly slower than the axenic pupae (*P* = 0.0024). In panel F, there were no statistically significant differences in the speed of development between samples for both pupa formation and adult eclosion. Download FIG S4, TIF file, 14.1 MB. Copyright © 2018 Sannino et al. 2018 Sannino et al. This content is distributed under the terms of the Creative Commons Attribution 4.0 International license.

### *A. pomorum* associates with *D. melanogaster* on chemically defined media.

To confirm that *A. pomorum* associated with the flies in our gnotobiotic studies, adults that developed from the eggs were homogenized and plated on MRS medium, and as expected, *A. pomorum* colonies were the only colonies recovered. In the polyassociation, *A. pomorum* was always recovered, with *L. brevis* colonies appearing frequently, and *L. plantarum* colonies occasionally appearing as well, though not as consistently as *L. brevis*. No *A. tropicalis* colonies were recovered.

Throughout the longevity experiments (from day 5 to the end of the experiment), recently deceased CR adults on the 0.2- and 0.00-µg/ml thiamine diets were homogenized and plated on MRS plates (Fig. S5A). In each instance, only *A. pomorum* was recovered from the flies. We also homogenized and plated the adults that developed from the eggs deposited at both 4 and 28 days. Again, the only microorganism recovered was *A. pomorum*. Our findings from the longevity experiment are consistent with the gnotobiotic experiments and show that on the no-thiamine diet, *A. pomorum* was selected for, as it is able to persist, associate with, and provide thiamine to its host throughout its life span, and its association was unrelated to the presence of dietary thiamine.

10.1128/mBio.00155-18.5 FIG S5  Recovery of bacteria from conventional and gnotobiotic flies on the no-thiamine diet and gnotobiotic fly development on the no-thiamine diet. (A) MRS agar plate inoculated with 100 µl of fly homogenate from flies grown on the no-thiamine diet. All colonies on the plate are *A. pomorum*. (B) MRS agar plate spiral plated with 50 µl of the homogenate of five adult gnotobiotic flies reassociated with the four bacterial species. There are two colony types present on the plate; the larger tan tinted colonies are *A. pomorum* (green arrow), while the smaller white colonies are *L. brevis* (red arrow). (C) Gnotobiotic fly development on the no-thiamine diet. The tube to the left depicts gnotobiotic flies monoassociated with *A. pomorum*. Pupae and adults are present. The tube to the right shows gnotobiotic flies associated with *L. brevis*, with only the dead melanized larvae visible. Download FIG S5, TIF file, 17.6 MB. Copyright © 2018 Sannino et al. 2018 Sannino et al. This content is distributed under the terms of the Creative Commons Attribution 4.0 International license.

### Wild-type *A. pasteurianus* rescues development, but a thiamine auxotroph does not.

To demonstrate that microbiota production of thiamine rescues development, we attempted to generate thiamine auxotrophic *A. pomorum* mutants. Unfortunately, all attempts to genetically manipulate *A. pomorum* failed. Instead we used its close relative *A. pasteurianus* SKU1108 (NBRC 101655), which was previously associated with *D. melanogaster* in comparative studies with *A. pomorum* (15, 18), as a proxy. This strain shares ~90% average percent nucleotide identity with *A. pomorum*, and it is capable of growing on the no-thiamine diet when preservatives were not present. Thiamine auxotrophs were generated through a deletion of the *thiC* gene, preventing production of hydroxymethyl pyrimidine, an essential precursor for thiamine synthesis (32). We found that when the wild-type strain was monoassociated with fly eggs, 34.9% of eggs developed into adults (Fig. 4C). The thiamine auxotroph was not able to rescue development, as no pupae or adults were generated (Fig. 4C and D) after association with fly eggs. Flies developed only when the thiamine production pathway of *A. pasteurianus* was intact, demonstrating that microbial thiamine production was solely responsible for the rescue.

### *A. pomorum* produces thiamine on the thiamine-free fly diet.

**FIG 5:**
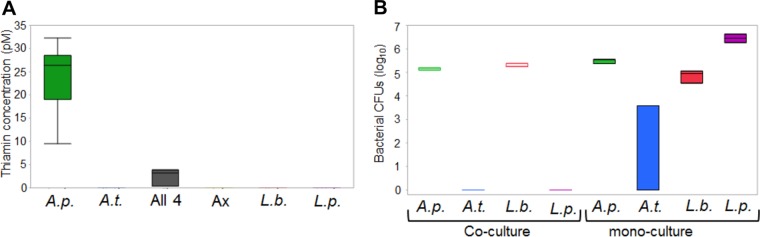
Production of thiamine and growth of the bacteria on the no-thiamine diet. (A) Thiamine assay results for the no-thiamine diet inoculated with *D. melanogaster* microbiota. Box plots represent the amount of thiamine produced on the no-thiamine diet by individual microbiota members or all four in concert. Thiamine concentrations are shown in picomoles per gram of food. (B) CFU recovered for each bacterium per gram of food at 3 days postinoculation. Coculture plots represent the CFU recovered of individual microbiota members from a mixed inoculum of all four microbiota members. Abbreviations: *A.p.*, *A. pomorum*; *A*.*t*., *A. tropicalis*; *L*.*b*., *L. brevis*; *L*.*p*., *L. plantarum*; All 4, all four microbiota members.

The results of our experiments suggest that *A. pomorum*, like *A. pasteurianus*, was directly transforming the 0.00-μg/ml thiamine diet through thiamine production, and this was rescuing fly development. To confirm this, we measured extracellular thiamine when bacteria were cultured on the thiamine-free food. At 6 days postinoculation, we did not recover thiamine from the negative control or from media inoculated with *A. tropicalis*, *L. brevis*, or *L. plantarum* (Fig. 5A). On average, media inoculated with *A. pomorum* yielded 24 pmol of thiamine per gram of food. When all four species were added in coculture, we recovered 10-fold-less thiamine, on average only 2.5 pmol per gram of food. The difference in thiamine recovered from the *A. pomorum* monoculture compared to the community culture likely contributes to the observed lower survival of polyassociation gnotobiotic flies (Fig. 4A and B). Additionally or alternatively, thiamine from the microbiota may be more available to developing flies than dietary thiamine, perhaps due to lysis of bacteria in the host intestine.

The discrepancies between the mono- and coculture thiamine concentrations may be due to growth of *L. brevis* in the coculture (Fig. 5B). Although *L. brevis* is able to grow on the food on its own, it was the only species able to grow with *A. pomorum*. In both mono- and polyassociation, there is no difference in growth of both species. It is possible that *A. pomorum* could be providing *L. brevis* with thiamine or another metabolic product, but this did not influence *L. brevis* growth (Fig. 5B). If *L. brevis* is using the thiamine produced by *A. pomorum*, this could reduce the total amount of thiamine available to the host. Both *A. tropicalis* and *L. plantarum* do not grow in the coculture; however, *L. plantarum* produces the highest number of CFU in monoculture, while *A. tropicalis* did not grow well in monoculture (Fig. 5B). Taken together, these data show that the thiamine produced by *A. pomorum* is the rescuing factor, as the other members of the microbiota can grow but do not produce measurable amounts of thiamine and do not rescue host development. Despite the potential for *A. pomorum* to provide thiamine for *L. brevis*, the fly developmental rescue is not contingent upon both species being present.

## DISCUSSION

In the present study, we investigated the tripartite interaction between *D. melanogaster*, its microbiota, and dietary thiamine. We demonstrated that the interaction of dietary thiamine and microbiota does not have a significant impact on the life history traits of CR adult flies, as providing them a thiamine-free diet does not reduce life span or their ability to produce eggs. We observed a microbiota effect on the life span of flies, as CR flies do not live as long as Ax flies, a finding that corroborates results from previous publications (22). The major finding of this study is the impact that the interaction between diet and microbiota has on the development of flies. Ax eggs laid on a thiamine-free diet are unable to pupate and died in the larval stage; however, the presence of the microbiota in CR flies rescues their development, demonstrating that the microbiota functions to provide thiamine to its host. This developmental rescue occurs both in eggs laid by young females and eggs laid by older females, indicating that the microbiota impacts the host-thiamine interaction throughout the host’s life span. By reassociating Ax eggs with one or multiple members of their indigenous microbiota, we were able to demonstrate that *Acetobacter pomorum* is the only microbiota member capable of rescuing development despite the genomic potential for other microbiota members to synthesize thiamine. Further, we found that it is the only member capable of producing thiamine on food. Using *A. pasteurianus* as a proxy, we showed that bacterial production of thiamine is solely responsible for the developmental rescue of the host.

Our finding that the dietary thiamine by microbiota interaction does not have a significant impact on *Drosophila* longevity likely has to do with how the adults were reared. The CR and Ax fly stocks were developed on standard medium containing yeast and cornmeal, and both of these components contain thiamine. The larval stores of thiamine carried into adulthood likely provided the adults with enough thiamine to survive, irrespective of their diet as adults. Similar results were found when assessing the impact of B vitamins on the longevity of the house fly *Musca domestica* grown axenically, where the absence of thiamine did not significantly reduce life span compared to those fed thiamine-replete diets (48). Other holometabolous adult insects have low-vitamin diets or do not eat at all, suggesting B vitamins are superfluous for them, and our results are consistent with this. In these insects, the critical period for acquiring B vitamins is the larval stage, as stores can support them in adulthood, which our study indicates. The lack of a thiamine-dependent impact on Ax female egg production is in direct contrast with what has been previously reported for *D. melanogaster*. Sang and King (41) determined that omitting dietary thiamine for 8 days reduced egg production by nearly 25% and 16 days on the diet completely eradicated production of viable eggs. Although we used different flies in our study, the major factor that likely contributed to our observed differences is the way in which the adults were raised. Sang and King raised their fly stocks on a semisynthetic diet already low in thiamine; therefore, larval stores in the egg-laying adults were likely low. The thiamine stores in flies used in our study were likely replete enough that after 4 weeks, egg output was not diminished.

The inability of Ax larvae to pupate on the no-thiamine diet demonstrates the critical nature of vitamin B_1_ in development. This finding is consistent with thiamine deficiencies that occur in natural populations of other animals such as alligators (49) and Great Lakes salmonids (50) that lead to death of progeny in the early stages of their development, suggesting that our system can serve as a potential model to investigate the factors that impact thiamine deficiency in other animals, including vertebrates. Unique to our study, we were able to demonstrate that the microbiota mitigates the developmental mortality caused by thiamine deficiency by supplying *Drosophila* with thiamine. The rescue of eggs laid by younger females on the no-thiamine diet was only partial, as it did not attain the same survivorship as when dietary vitamin B_1_ was provided but still allowed for pupation and emergence of adults. When dietary thiamine was provided, the microbiota enhanced both development speed and survivorship (Fig. 3A and B). The microbiota exerted a greater influence on the eggs laid by older females, as CR flies on the no-thiamine diet did not have significantly lower survival (compare Fig. 3A to C). This improved survivorship on the no-thiamine diet is likely due to the increase in bacterial load that typically occurs with flies as they age (3, 11). As flies age, the composition of their microbiota shifts, with older flies having communities dominated by *A. pomorum* (5). The higher titers of *A. pomorum* at 4 weeks likely had a twofold effect. (i) Mothers were able to provision more thiamine to their eggs due to the increased numbers of this thiamine-producing microbiota member. (ii) Mothers were able to seed the food with a higher abundance of *A. pomorum* to exert direct influence on eggs and larvae through thiamine production. This is likely how the bacteria were able to exert influence. Vectoring the microbiota to a food source to serve as a vitamin reservoir may be a strategy used by species of *Drosophila*. In the wild, many *Drosophila* species feed on overripe or damaged fruits, which have a high yeast content; however, *Drosophila suzukii* is an invasive pest which feeds on soft, marketable fruit (51). It is possible that *D. suzukii* relies on its microbiota, which it vectors to the fruit, for the supply of nutrients such as thiamine. If this holds true, targeting the microbiota may be an effective approach for pest management.

Previous studies have implicated the microbiota in providing B vitamins such as riboflavin (9, 13) and folate (14, 25) to *Drosophila*, however, this current study is the first to depict the provision of thiamine. Wong and colleagues (13) may have failed to see a thiamine-microbiota response in their study due to the use of undefined diets containing yeast, which are able to supply excess thiamine to the host. Our results establish that *A. pomorum* is the symbiont responsible for thiamine provision to *D. melanogaster*, and this is comparable to the rescue observed for CR flies on the no-thiamine diet at 4 days. Like the CR flies on the no-thiamine diet, this rescue is only partial. It is likely that under different conditions, when *A. pomorum* and other thiamine-producing microbiota members proliferate more, thiamine production would have a greater influence on the host. Gnotobiotic studies show that development of eggs on a no-thiamine diet is rescued only when *A. pomorum* is reassociated with Ax eggs. This is congruent with the genomic potential of *A. pomorum*, as it has a complete pathway for *de novo* thiamine biosynthesis (15). Unlike the lactobacilli, *A. tropicalis* also has a complete set of genes for thiamine biosynthesis (15), but it was not able to grow to high densities on the food and produce thiamine. It is possible that on a different diet or in other circumstances, it too could provide thiamine for the host, but in this experiment, *A. pomorum* was selected for by this particular diet. The lactobacilli may have been able to persist on the food because they lack a complete tricarboxylic acid (TCA) cycle (15) and consequently may have a reduced need for thiamine. Further, we confirmed that *A. pomorum* produces thiamine on the no-thiamine diet. This is another example of an *Acetobacter* species manipulating the host diet leading to an impact on host health (18, 19). It is unclear whether extracellular thiamine detected in the fly food was released by *A. pomorum* cell lysis while the bacteria grew on the food or whether it was due to the efflux of thiamine from the cell. The extracellular thiamine produced by *A. pomorum* was much lower than the thiamine present in the lowest thiamine-containing diet. Vitamin B_1_ provision may also occur in the gut of *Drosophila* either by bacteria lysing in the gut, or thiamine production inside the gut. Our assay for measuring extracellular thiamine relied on diffusion from solid media, and could have resulted in an underestimation of the total extracellular thiamine produced by *A. pomorum*. More experiments are need to determine the exact mechanisms of how the host is acquiring thiamine from *A. pomorum*. By using *A. pasteurianus*, we were able to show that when the ability to make thiamine is prevented, the rescue of development does not occur depicting that microbiota thiamine production is responsible for the rescue. When a genetic tool is established for *A. pomorum*, we expect thiamine auxotroph mutants will not rescue development as well.

*A. pomorum* has already been recognized as an important member of the microbiota, as it regulates host signaling, affecting fly development and body size (6). It is important in maintaining host nutritional indexes as flies associated with *A. pomorum* have lower triacylglycerol (TAG) levels than Ax flies (16). Despite being the first symbiont shown to produce thiamine for *Drosophila*, it is not the first insect symbiont shown to produce thiamine for its host. *Rhodococcus rhodnii*, the extracellular symbiont of the Chagas disease vector *Rhodnius prolixus*, produces thiamine in sterile horse blood, allowing for the growth and development of its host (52). *Wigglesworthia glossinidia* and *Sodalis glossinidius* are two enteric symbionts of the tsetse fly, and in the fly, *Wigglesworthia* produces thiamine that is used by *Sodalis*. Promotion of the growth of *Sodalis* maintains host gut homeostasis and health (53, 54). This promotion of host health through interactions with other community members does not appear to be occurring in our system as the polyassociated gnotobiotic flies have reduced survival compared to the *A. pomorum*-monoassociated flies. The presence of *L. brevis* may be deleterious; less thiamine is produced when it is present, and survival is reduced in monoassociated flies when dietary thiamine is present. *A. pomorum* abundance is similar in the monocultures and cocultures (Fig. 5B), suggesting that the presence of *L. brevis* accounts for the differences in thiamine production. This implies there may be competition between host and auxotrophic microbiota members for thiamine, resulting in the reduction of survival in the polyassociation gnotobiotic flies.

The evolution of symbiosis is not well understood; what is the reckoning of costs and benefits of microbial association? Our study gives important mechanistic insight toward answering this question. Specifically, it contributes to the growing body of evidence that in *Drosophila*, microbes play a major role in vitamin provision. Our data indicate that, while the microbiota is deleterious for long-term health, measured by adult longevity, it is essential for the development of offspring in low-thiamine dietary contexts. Thus, the microbiota buffers fly fitness against dietary deficiency but limits life span. Altogether, these data indicate that the interaction of dietary thiamine and microbiota is a mechanism of a life span-reproduction tradeoff.

In conclusion, we show that *D. melanogaster* microbiota, in particular *A. pomorum*, is an essential thiamine supplier in low-thiamine conditions. It transforms nutrition through thiamine production, rescuing the development of axenic flies in the absence of dietary thiamine. This demonstrates the importance of the microbiota as nutritional symbionts and shows how certain environmental conditions may select for microbiota to produce specific nutrients for the host. Our study provides a robust system to further interrogate the relationship between *D. melanogaster*, its microbiota, and other dietary components.

## MATERIALS AND METHODS

### *Drosophila* and bacterial stocks.

Throughout this study, *Wolbachia*-free Canton^S^ flies were used. Conventionally raised (CR) laboratory stocks were cultured on a standard diet containing 5% (wt/vol) yeast, 4% (wt/vol) glucose, and 6% (wt/vol) cornmeal at 25°C. Axenic (Ax) stocks were maintained on a sterile diet containing 5% (wt/vol) yeast, 4% (wt/vol) sucrose, and 6% (wt/vol) cornmeal in sterile glass tubes at 25°C (3). For each passage, the Ax stocks were transferred aseptically into new tubes. After each transfer, the flies were removed from the food after 2 days of egg laying, at least 5 flies/tube were put into sterile microcentrifuge tubes containing 500 µl of sterile 1× phosphate-buffered saline (PBS) and homogenized, and the homogenate was plated on MRS agar to ensure that conditions were axenic. The plates were incubated at 29°C, and if there was any growth, the corresponding tube of flies was discarded. *Acetobacter pomorum* DmCS_004, *Acetobacter tropicalis* DmCS_006, *Lactobacillus brevis* DmCS_003, *Lactobacillus plantarum* DmCS_001 (15), and *Acetobacter pasteurianus* SKU1108 (NBRC 101655) (55) were used in this study. *Acetobacter* spp. were grown at 30°C on MRS plates in aerobic conditions, while the *Lactobacillus* spp. were grown on MRS at 30°C in an atmosphere of 95% CO_2_ and 5% H_2_ in a Coy vinyl anaerobic chamber (Coy Laboratory Products, Inc.). When grown in liquid MRS, the *Acetobacter* spp. were grown with shaking at 225 rpm, while the *Lactobacillus* spp. were grown without shaking.

### Generation of *A. pasteurianus* SKU1108 *thiC* mutant.

A PCR fragment of the upstream and downstream regions of the *thiC* open reading frame (ORF) was inserted into the pK18molGII vector to generate the pK18molGII-Δ*thiC* construct. *A. pasteurianus* SKU1108 was transformed with pK18molGII-Δ*thiC* by a triparental mating method using *Escherichia coli* HB101/pKR2013 (56). Resulting colonies with β-glucuronidase-positive and kanamycin-resistant phenotypes were selected to perform the second recombination. Colonies with β-glucuronidase-negative and kanamycin-sensitive phenotypes were then selected. The deleted region (from amino acids 6 to 622 of ThiC) in these recombinant clones was confirmed by PCR analysis and sequencing of the PCR products.

### Generation of axenic and gnotobiotic flies.

Ax flies were produced as previously described (16) with one amendment; we used 10% bleach (vol/vol) for egg dechorionation. The same protocol was followed to produce gnotobiotic flies with some modifications. Overnight cultures were pelleted and resuspended in a thiamine-free, chemically defined medium (DM4) to a final density of 10^8^ cells/ml, using the empirically derived constants for each species (16). DM4 is based on M9 medium (57), but it is buffered by 0.1 M morpholineethanesulfonic acid (MES), pH 6.0. DM4 contains 10 mM FeSO_4_, 9.5 mM NH_4_Cl, 0.276 mM K_2_SO_4_, 0.5 µM CaCl_2_, 0.525 mM MgCl_2_, 50 mM NaCl, 1.32 mM K_2_HPO_4_, 1% (vol/vol) vitamin supplement (ATCC MDVS), 1% (vol/vol) trace mineral solution (ATCC MD-TMS), 0.1% (wt/vol) glucose, and 12.5% (vol/vol) amino acid mix based on published concentrations (58). Cell pellets were washed two times with DM4 to remove thiamine carryover. For generating monoassociated flies, 5 × 10^6^ cells/vial were added to the food surface containing the sterile eggs. When all four species were reassociated, 1.25 × 10^6^ cells/vial of each species were added. Sterile DM4 was added to eggs to serve as the Ax negative control. *A. pasteurianus*-associated gnotobiotic flies were generated on the no-thiamine media lacking preservatives. To generate the eggs for the gnotobiotic experiments, 20 Ax females and 5 Ax males were transferred aseptically from standard medium to each tube of chemically defined diet and laid eggs for ≤18 h.

### Chemically defined fly diet.

For all experiments, we used a previously described defined diet (25) with a few minor alterations. Thiamine adsorbs to glassware (59) so precautions were taken to prevent thiamine contamination. When autoclaving was not needed, plasticware was used, but when glassware was necessary, it was washed with Fisherbrand cleaning solution (catalog no. SC88-500; Fisher Scientific), rinsed 10 times with tap water and then 10 times with deionized H_2_O, and baked overnight at ~200°C. Since thiamine is base labile, a 0.1 M NaOH wash was conducted, followed by the same rinsing and baking procedure. The only amendments to the media were that 0.3 g/liter cholesterol and 0.5 g/liter l-cysteine HCl were added. Four diets were generated containing 0.00, 0.04, 0.2, or 1.0 µg/ml thiamine.

### Longevity experiments.

Three-day-old CR flies (20 females and 5 males) were transferred to each sterile diet tube. For each experiment, there were three tubes set up for each diet, and this experiment was repeated three times for a total of 225 flies per diet. The same design was used for Ax flies; however, all transfers were conducted aseptically in a sanitized SterileGARD biosafety cabinet. All flies were transferred to new sterile food every 3 or 4 days (CR flies were anesthetized with CO_2_ for passage, while Ax flies were anesthetized on ice to maintain sterility). The tubes were incubated at 25°C on a 12-h light/12-h dark cycle and checked daily. To monitor sterility in Ax flies throughout the experiment, individual dead flies in each of the vials were collected aseptically and screened for microbial growth as described above. The experiments were run until every fly died, and the data for each experiment were pooled and analyzed via a Cox proportional hazards (CoxPH) model in SAS 9.4. The presence of microbiota, thiamine concentration, the thiamine concentration × microbiota interaction term, and replicate were fixed effects, with the tube in each experiment being a random effect. Ninety-five percent Wald confidence limits (CL) and point estimates were used to determine the significance of each comparison. Significant differences do not have a proportion of 1 within their CL.

### Egg output experiments.

Ax and CR flies were transferred to new sterile food at days 4 and 28 of the experiment where they laid eggs for ≤18 h. The eggs were counted for each tube, and the average number of eggs/female was calculated and analyzed using a fit model, which includes an analysis of variance (ANOVA) in JMP Pro 12.0.1. The presence of microbiota, thiamine concentration, replicate, and the vitamin B_1_ concentration × microbiota cross were all fixed effects. The average numbers of eggs per female were log transformed and used as the input data. If there was a statistically significant value from the ANOVA, pairwise comparisons were made using a *t* test, and *P* values were adjusted by Bonferroni’s correction as 28 comparisons were made.

### Insect development.

Experiments were conducted to assess the impact of dietary thiamine and microbiota on fly development. For the diet experiments, the eggs laid for the egg output experiments proceeded to develop for each diet at both egg-laying periods. There were three tubes for each experiment, which was replicated three times. For the gnotobiotic experiments, there were three tubes for two replicates per bacterial addition on the 0.2-µg/ml thiamine diet. For the no-thiamine diet, there were two replicates with three tubes per replicate for the addition of *L. brevis*, *L. plantarum*, and *A. tropicalis*, and three replicates for the Ax flies, *A. pomorum*, and all four species addition treatments.

The tubes were checked daily, and progression of the larvae was scored. The number of pupae and subsequently adults in each tube was recorded. The data for replicates were pooled, and developmental speed was plotted as the average number of days to reach 100% pupation and 100% adult emergence. The time for 50% of all flies to reach pupation and adulthood was determined for each tube using the slope of the development rates. These values were analyzed using a fit model in JMP 12.0.1 which included an ANOVA, with treatment and replicate as the fixed effects. Confidence intervals were checked to ensure that they did not include zero. If the ANOVA was significant, pairwise comparisons were made via a *t* test. *P* values for the diet by microbiota experiments were adjusted by Bonferroni’s correction for the 21 comparisons. *P* values for the gnotobiotic fly experiments on the 0.2-µg/ml thiamine diet were adjusted by Bonferroni’s correction for 15 comparisons.

### Survival analysis of development studies.

Development survival data were analyzed in R 3.4.0 using Cox proportional hazards models from the rms library. Time to development was modeled as a function of experimental condition and replicate, both as unordered factors. Conditions in which no flies developed were excluded from the analyses. For scoring pupa development, those that did not develop by 24 days were excluded. For egg development to adulthood, in all the experiments, those that did not develop to adults by 28 days were excluded. Tukey *posthoc* comparisons were conducted in R 3.4.0 using the multcomp library to identify pairwise differences between conditions. *P* values were adjusted by Bonferroni’s correction for multiple comparisons (the development experiments of the eggs laid at  days 4 and 28 were corrected for 21 comparisons, and the 0.2-μg/ml diet gnotobiotic experiments were corrected for 15 comparisons).

### Microbial identification.

Throughout the longevity experiments, dead flies on the 0.2- and 0.00-µg/ml thiamine diets were collected aseptically after the living flies were transferred to new sterile food. Individual flies were homogenized and plated on MRS agar plates as described above. Colonies were visually inspected and categorized on the basis of morphology. Colony PCRs were conducted on multiple colonies using the 8F and 1492R universal 16S rRNA gene primers (60), and the PCR products were sequenced using the Sanger method at the Cornell University Institute of Biotechnology. Sequences were subjected to a BLAST search to determine the identity of the bacteria and aligned to reference 16S rRNA gene sequences from our fly microbiota isolates using Geneious 6.0.6. To determine the identity of the members of the microbiota of the developing flies, five 1- to 2-day-old adults were pooled, homogenized, and plated on MRS agar as described above. Colonies were sequenced to confirm their identity. The same plating technique was used to confirm the bacteria associated with the flies in the gnotobiotic experiments. In the polyassociation studies, homogenate was plated on MRS agar and incubated anoxically to select for *Lactobacillus* spp. or in the presence of oxygen and 5 µg/ml ampicillin to prevent growth of the lactobacilli. Colony morphology was used to identify the bacteria (16).

### Measurement of thiamine levels.

The bacteria were added to ~7.5 ml of the no-thiamine food in 50-ml sterile Falcon tubes in the same manner as for the gnotobiotic experiments. Three tubes were used for each treatment except for the *A. pomorum* treatment which had six tubes. The tubes were inspected under a dissecting microscope daily for growth. At 6 days postinoculation, the samples were processed. The concentrations of thiamine produced in the fly food media were assessed using modifications to a previously reported competitive binding assay specific for thiamine, and plates were prepared as detailed previously (61). The solid fly medium was diluted at a 20% (wt/vol) ratio with 20/200 mM MESS (20 mM MES [pH 6.5], 200 mM sodium chloride) and incubated at ambient temperature for 5 h with vortexing. The suspensions were centrifuged at 2,500 rpm for 5 min. Two hundred microliters of supernatant was diluted with 200 μl of 900 mM MES and 200 mM NaCl (pH 6.6), and the samples were thoroughly vortexed. Aliquots (50 µl) of supernatant were added in quadruplicate to the washed wells on the microtiter plate. Thiamine standards were prepared in the same manner using media without bacteria. Thiamine binding protein-conjugated liposomes were then added to the plate and mixed as detailed previously (61). The plate was processed, and fluorescence measurements and data analysis were performed as described previously (61). The critical modification to the previously reported method was the marked increase to 900 mM MES to adequately buffer the pH change in the medium due to the acetic acid produced by *A. pomorum*. The assay performs optimally at a pH value of ~6.5, whereas the pH of the medium in the presence of *A. pomorum* was 4.4 under the original 20 mM buffer conditions. Thiamine concentration was corrected for based on buffer volume and determined per gram of food.

### Bacterial growth on the chemically defined diets.

The bacteria were grown on the no-thiamine diet as described above for the thiamine quantification experiment, with three tubes for each bacterial inoculation. Growth was inspected under a dissecting microscope daily. At 3 days, 3 ml of 1× PBS was added directly to the surface of the food. Using a Gilson P1000 pipette, the PBS was mixed by vigorously pipetting up and down. One milliliter was removed and put into a sterile microcentrifuge tube, and 1/100 and 1/1000 dilutions were made and plated using a WASP2 spiral plater (Microbiology International). The monocultures and cocultures were plated as described in the gnotobiotic section. Colonies were counted, and the number of CFU per milliliter of medium was determined.

## References

[1] McFall-Ngai M, Hadfield MG, Bosch TC, Carey HV, Domazet-Lošo T, Douglas AE, Dubilier N, Eberl G, Fukami T, Gilbert SF, Hentschel U, King N, Kjelleberg S, Knoll AH, Kremer N, Mazmanian SK, Metcalf JL, Nealson K, Pierce NE, Rawls JF, Reid A, Ruby EG, Rumpho M, Sanders JG, Tautz D, Wernegreen JJ 2013 Animals in a bacterial world, a new imperative for the life sciences. Proc Natl Acad Sci U S A 110:3229–3236. doi:10.1073/pnas.1218525110. 23391737 PMC3587249

[2] Liu X, Hodgson JJ, Buchon N 2017 Drosophila as a model for homeostatic, antibacterial, and antiviral mechanisms in the gut. PLoS Pathog 13:e1006277. doi:10.1371/journal.ppat.1006277. 28472194 PMC5417715

[3] Broderick NA, Buchon N, Lemaitre B 2014 Microbiota-induced changes in *Drosophila melanogaster* host gene expression and gut morphology. mBio 5:e01117-14. doi:10.1128/mBio.01117-14. PMC4045073 24865556

[4] Wong AC, Chaston JM, Douglas AE 2013 The inconstant gut microbiota of *Drosophila* species revealed by 16S rRNA gene analysis. ISME J 7:1922–1932. doi:10.1038/ismej.2013.86. 23719154 PMC3965314

[5] Wong CNA, Ng P, Douglas AE 2011 Low-diversity bacterial community in the gut of the fruit fly *Drosophila melanogaster*. Environ Microbiol 13:1889–1900. doi:10.1111/j.1462-2920.2011.02511.x. 21631690 PMC3495270

[6] Shin SC, Kim S-H, You H, Kim B, Kim AC, Lee K-A, Yoon J-H, Ryu J-H, Lee W-J 2011 *Drosophila* microbiome modulates host developmental and metabolic homeostasis via insulin signaling. Science 334:670–674. doi:10.1126/science.1212782. 22053049

[7] Storelli G, Defaye A, Erkosar B, Hols P, Royet J, Leulier F 2011 *Lactobacillus plantarum* promotes *Drosophila* systemic growth by modulating hormonal signals through TOR-dependent nutrient sensing. Cell Metab 14:403–414. doi:10.1016/j.cmet.2011.07.012. 21907145

[8] Ren C, Webster P, Finkel SE, Tower J 2007 Increased internal and external bacterial load during *Drosophila* aging without life span trade-off. Cell Metab 6:144–152. doi:10.1016/j.cmet.2007.06.006. 17681150

[9] Fridmann-Sirkis Y, Stern S, Elgart M, Galili M, Zeisel A, Shental N, Soen Y 2014 Delayed development induced by toxicity to the host can be inherited by a bacterial-dependent, transgenerational effect. Front Genet 5:27. doi:10.3389/fgene.2014.00027. 24611070 PMC3933808

[10] Ryu J-H, Kim S-H, Lee H-Y, Bai JY, Nam Y-D, Bae J-W, Lee DG, Shin SC, Ha E-M, Lee W-J 2008 Innate immune homeostasis by the homeobox gene caudal and commensal-gut mutualism in *Drosophila*. Science 319:777–782. doi:10.1126/science.1149357. 18218863

[11] Buchon N, Broderick NA, Chakrabarti S, Lemaitre B 2009 Invasive and indigenous microbiota impact intestinal stem cell activity through multiple pathways in *Drosophila*. Genes Dev 23:2333–2344. doi:10.1101/gad.1827009. 19797770 PMC2758745

[12] Sharon G, Segal D, Ringo JM, Hefetz A, Zilber-Rosenberg I, Rosenberg E 2010 Commensal bacteria play a role in mating preference of *Drosophila melanogaster*. Proc Natl Acad Sci U S A 107:20051–20056. doi:10.1073/pnas.1009906107. 21041648 PMC2993361

[13] Wong AC-N, Dobson AJ, Douglas AE 2014 Gut microbiota dictates the metabolic response of *Drosophila* to diet. J Exp Biol 217:1894–1901. doi:10.1242/jeb.101725. 24577449 PMC4037322

[14] Blatch SA, Meyer KW, Harrison JF 2010 Effects of dietary folic acid level and symbiotic folate production on fitness and development in the fruit fly *Drosophila melanogaster*. Fly 4:312–319. doi:10.4161/fly.4.4.13258. 20855945

[15] Newell PD, Chaston JM, Wang Y, Winans NJ, Sannino DR, Wong AC, Dobson AJ, Kagle J, Douglas AE 2014 In vivo function and comparative genomic analyses of the *Drosophila* gut microbiota identify candidate symbiosis factors. Front Microbiol 5:576. doi:10.3389/fmicb.2014.00576. 25408687 PMC4219406

[16] Newell PD, Douglas AE 2014 Interspecies interactions determine the impact of the gut microbiota on nutrient allocation in *Drosophila melanogaster*. Appl Environ Microbiol 80:788–796. doi:10.1128/AEM.02742-13. 24242251 PMC3911109

[17] Chaston JM, Dobson AJ, Newell PD, Douglas AE 2015 Host genetic control of the microbiota mediates the *Drosophila* nutritional phenotype. Appl Environ Microbiol 82:671–679. doi:10.1128/AEM.03301-15. 26567306 PMC4711117

[18] Chaston JM, Newell PD, Douglas AE 2014 Metagenome-wide association of microbial determinants of host phenotype in *Drosophila melanogaster*. mBio 5:e01631-14. doi:10.1128/mBio.01631-14. 25271286 PMC4196228

[19] Huang J-H, Douglas AE 2015 Consumption of dietary sugar by gut bacteria determines *Drosophila* lipid content. Biol Lett 11:20150469. doi:10.1098/rsbl.2015.0469. PMC4614424 26382071

[20] Guo L, Karpac J, Tran SL, Jasper H 2014 PGRP-SC2 promotes gut immune homeostasis to limit commensal dysbiosis and extend lifespan. Cell 156:109–122. doi:10.1016/j.cell.2013.12.018. 24439372 PMC3928474

[21] Buchon N, Broderick NA, Poidevin M, Pradervand S, Lemaitre B 2009 Drosophila intestinal response to bacterial infection: activation of host defense and stem cell proliferation. Cell Host Microbe 5:200–211. doi:10.1016/j.chom.2009.01.003. 19218090

[22] Clark RI, Salazar A, Yamada R, Fitz-Gibbon S, Morselli M, Alcaraz J, Rana A, Rera M, Pellegrini M, Ja WW, Walker DW 2015 Distinct shifts in microbiota composition during *Drosophila* aging impair intestinal function and drive mortality. Cell Rep 12:1656–1667. doi:10.1016/j.celrep.2015.08.004. 26321641 PMC4565751

[23] Biteau B, Hochmuth CE, Jasper H 2008 JNK activity in somatic stem cells causes loss of tissue homeostasis in the aging *Drosophila* gut. Cell Stem Cell 3:442–455. doi:10.1016/j.stem.2008.07.024. 18940735 PMC3225008

[24] LeBlanc JG, Milani C, de Giori GS, Sesma F, Van Sinderen D, Ventura M 2013 Bacteria as vitamin suppliers to their host: a gut microbiota perspective. Curr Opin Biotechnol 24:160–168. doi:10.1016/j.copbio.2012.08.005. 22940212

[25] Piper MD, Blanc E, Leitão-Gonçalves R, Yang M, He X, Linford NJ, Hoddinott MP, Hopfen C, Soultoukis GA, Niemeyer C, Kerr F, Pletcher SD, Ribeiro C, Partridge L 2014 A holidic medium for *Drosophila melanogaster*. Nat Methods 11:100–105. doi:10.1038/nmeth.2731. 24240321 PMC3877687

[26] Nabokina SM, Inoue K, Subramanian VS, Valle JE, Yuasa H, Said HM 2014 Molecular identification and functional characterization of the human colonic thiamine pyrophosphate transporter. J Biol Chem 289:4405–4416. doi:10.1074/jbc.M113.528257. 24379411 PMC3924303

[27] Carini P, Campbell EO, Morré J, Sañudo-Wilhelmy SA, Thrash JC, Bennett SE, Temperton B, Begley T, Giovannoni SJ 2014 Discovery of a SAR11 growth requirement for thiamin’s pyrimidine precursor and its distribution in the Sargasso Sea. ISME J 8:1727–1738. doi:10.1038/ismej.2014.61. 24781899 PMC4817611

[28] Sechi G, Sechi E, Fois C, Kumar N 2016 Advances in clinical determinants and neurological manifestations of B vitamin deficiency in adults. Nutr Rev 74:281–300. doi:10.1093/nutrit/nuv107. 27034475

[29] Balk L, Hägerroth P-Å, Åkerman G, Hanson M, Tjärnlund U, Hansson T, Hallgrimsson GT, Zebühr Y, Broman D, Mörner T, Sundberg H 2009 Wild birds of declining European species are dying from a thiamine deficiency syndrome. Proc Natl Acad Sci U S A 106:12001–12006. doi:10.1073/pnas.0902903106. 19597145 PMC2715476

[30] Balk L, Hägerroth P-Å, Gustavsson H, Sigg L, Åkerman G, Ruiz Muñoz YR, Honeyfield DC, Tjärnlund U, Oliveira K, Ström K, McCormick SD, Karlsson S, Ström M, van Manen M, Berg AL, Halldórsson HP, Strömquist J, Collier TK, Börjeson H, Mörner T, Hansson T 2016 Widespread episodic thiamine deficiency in Northern Hemisphere wildlife. Sci Rep 6:38821. doi:10.1038/srep38821. 27958327 PMC5153840

[31] Edwin EE, Jackman R 1970 Thiaminase I in the development of cerebrocortical necrosis in sheep and cattle. Nature 228:772–774. doi:10.1038/228772a0. 5528786

[32] Jurgenson CT, Begley TP, Ealick SE 2009 The structural and biochemical foundations of thiamin biosynthesis. Annu Rev Biochem 78:569–603. doi:10.1146/annurev.biochem.78.072407.102340. 19348578 PMC6078420

[33] Breves G, Hoeller H, Harmeyer J, Martens H 1979 Gastro-intestinal passage and balance of thiamin in sheep. Ann Rech Vet 10:465–466. 533199

[34] Breves G, Hoeller H, Harmeyer J, Martens H 1980 Thiamin balance in the gastrointestinal tract of sheep. J Anim Sci 51:1177–1181. doi:10.2527/jas1980.5151177x. 7204267

[35] Breves G, Brandt M, Hoeller H, Rohr K 1981 Flow of thiamin to the duodenum in dairy cows fed different rations. J Agric Sci 96:587–591. doi:10.1017/S0021859600034559.

[36] Said HM 2011 Intestinal absorption of water-soluble vitamins in health and disease. Biochem J 437:357–372. doi:10.1042/BJ20110326. 21749321 PMC4049159

[37] Arumugam M, Raes J, Pelletier E, Le Paslier D, Yamada T, Mende DR, Fernandes GR, Tap J, Bruls T, Batto J-M, MetaHIT Consortium 2011 Enterotypes of the human gut microbiome. Nature 473:174–180. doi:10.1038/nature09944. 21508958 PMC3728647

[38] Magnúsdóttir S, Ravcheev D, de Crécy-Lagard V, Thiele I 2015 Systematic genome assessment of B-vitamin biosynthesis suggests co-operation among gut microbes. Front Genet 6:148. doi:10.3389/fgene.2015.00148. 25941533 PMC4403557

[39] Bakula M 1969 The persistence of a microbial flora during postembryogenesis of *Drosophila melanogaster*. J Invertebr Pathol 14:365–374. doi:10.1016/0022-2011(69)90163-3. 4904970

[40] Leitão-Gonçalves R, Carvalho-Santos Z, Francisco AP, Fioreze GT, Anjos M, Baltazar C, Elias AP, Itskov PM, Piper MDW, Ribeiro C 2017 Commensal bacteria and essential amino acids control food choice behavior and reproduction. PLoS Biol 15:e2000862. doi:10.1371/journal.pbio.2000862. 28441450 PMC5404834

[41] Sang JH, King RC 1961 Nutritional requirements of axenically cultured *Drosophila melanogaster* adults. J Exp Biol 38:793–809.

[42] Lukienko PI, Mel’nichenko NG, Zverinskii IV, Zabrodskaya SV 2000 Antioxidant properties of thiamine. Bull Exp Biol Med 130:874–876. doi:10.1007/BF02682257. 11177269

[43] Iwata H 1982 Possible role of thiamine in the nervous system. Trends Pharmacol Sci 3:171–173. doi:10.1016/0165-6147(82)91074-4.

[44] Schwenke RA, Lazzaro BP, Wolfner MF 2016 Reproduction-immunity trade-offs in insects. Annu Rev Entomol 61:239–256. doi:10.1146/annurev-ento-010715-023924. 26667271 PMC5231921

[45] Clifton ME, Noriega FG 2011 Nutrient limitation results in juvenile hormone-mediated resorption of previtellogenic ovarian follicles in mosquitoes. J Insect Physiol 57:1274–1281. doi:10.1016/j.jinsphys.2011.06.002. 21708165 PMC3167010

[46] Sang JH 1956 The quantitative nutritional requirements of *Drosophila melanogaster*. J Exp Biol 33:45–72.

[47] Robertson FW, Sang JH 1944 The ecological determinants of population growth in a *Drosophila* culture. I. Fecundity of adult flies. Proc R Soc Lond B Biol Sci 132:258–277. doi:10.1098/rspb.1944.0017.

[48] Greenberg B, Burkman AM 1963 Effect of B-vitamins and a mixed flora on the longevity of germ-free adult houseflies, *Musca domestica* L. J Cell Comp Physiol 62:17–22. doi:10.1002/jcp.1030620104. 14054888

[49] Sepúlveda MS, Wiebe JJ, Honeyfield DC, Rauschenberger HR, Hinterkopf JP, Johnson WE, Gross TS 2004 Organochlorine pesticides and thiamine in eggs of largemouth bass and American alligators and their relationship with early life-stage mortality. J Wildl Dis 40:782–786. doi:10.7589/0090-3558-40.4.782. 15650100

[50] Honeyfield DC, Hinterkopf JP, Fitzsimons JD, Tillitt DE, Zajicek JL, Brown SB 2005 Development of thiamine deficiencies and early mortality syndrome in lake trout by feeding experimental and feral fish diets containing thiaminase. J Aquat Anim Health 17:4–12. doi:10.1577/H03-078.1.

[51] Hamby KA, Hernández A, Boundy-Mills K, Zalom FG 2012 Associations of yeasts with spotted-wing *Drosophila* (*Drosophila suzukii*; Diptera: Drosophilidae) in cherries and raspberries. Appl Environ Microbiol 78:4869–4873. doi:10.1128/AEM.00841-12. 22582060 PMC3416361

[52] Harington JS 1960 Synthesis of thiamine and folic acid by *Nocardia rhodnii*, the micro-symbiont of *Rhodnius prolixus*. Nature 188:1027–1028. doi:10.1038/1881027a0. 13711570

[53] Snyder AK, Deberry JW, Runyen-Janecky L, Rio RV 2010 Nutrient provisioning facilitates homeostasis between tsetse fly (Diptera: Glossinidae) symbionts. Proc Biol Sci 277:2389–2397. doi:10.1098/rspb.2010.0364. 20356887 PMC2894912

[54] Snyder AK, McLain C, Rio RV 2012 The tsetse fly obligate mutualist Wigglesworthia morsitans alters gene expression and population density via exogenous nutrient provisioning. Appl Environ Microbiol 78:7792–7797. doi:10.1128/AEM.02052-12. 22904061 PMC3485737

[55] Matsutani M, Hirakawa H, Saichana N, Soemphol W, Yakushi T, Matsushita K 2012 Genome-wide phylogenetic analysis of differences in thermotolerance among closely related Acetobacter pasteurianus strains. Microbiology 158:229–239. doi:10.1099/mic.0.052134-0. 22016572

[56] Figurski DH, Helinski DR 1979 Replication of an origin-containing derivative of plasmid RK2 dependent on a plasmid function provided in trans. Proc Natl Acad Sci U S A 76:1648–1652. 377280 10.1073/pnas.76.4.1648 PMC383447

[57] Neidhardt FC, Bloch PL, Smith DF 1974 Culture medium for enterobacteria. J Bacteriol 119:736–747. 4604283 10.1128/jb.119.3.736-747.1974 PMC245675

[58] Harwood CR, Cutting SM 1990 Molecular biological methods for *Bacillus*. John Wiley, Chichester, United Kingdom.

[59] Farrer K, Hollenberg W 1953 Adsorption of thiamine on glassware. Analyst 78:730–731.

[60] Turner S, Pryer KM, Miao VP, Palmer JD 1999 Investigating deep phylogenetic relationships among cyanobacteria and plastids by small subunit rRNA sequence analysis. J Eukaryot Microbiol 46:327–338. doi:10.1111/j.1550-7408.1999.tb04612.x. 10461381

[61] Edwards KA, Seog WJ, Han L, Feder S, Kraft CE, Baeumner AJ 2016 High-throughput detection of thiamine using periplasmic binding protein-based biorecognition. Anal Chem 88:8248–8256. doi:10.1021/acs.analchem.6b02092. 27460839

