# Supplementary figures and images for: The Drosophila melanogaster Gut Microbiota Provisions Thiamine to Its Host
Source: mBio. 2018 Mar 6;9(2):e00155-18. doi: 10.1128/mBio.00155-18 (PMC5845000; doi:10.1128/mBio.00155-18)

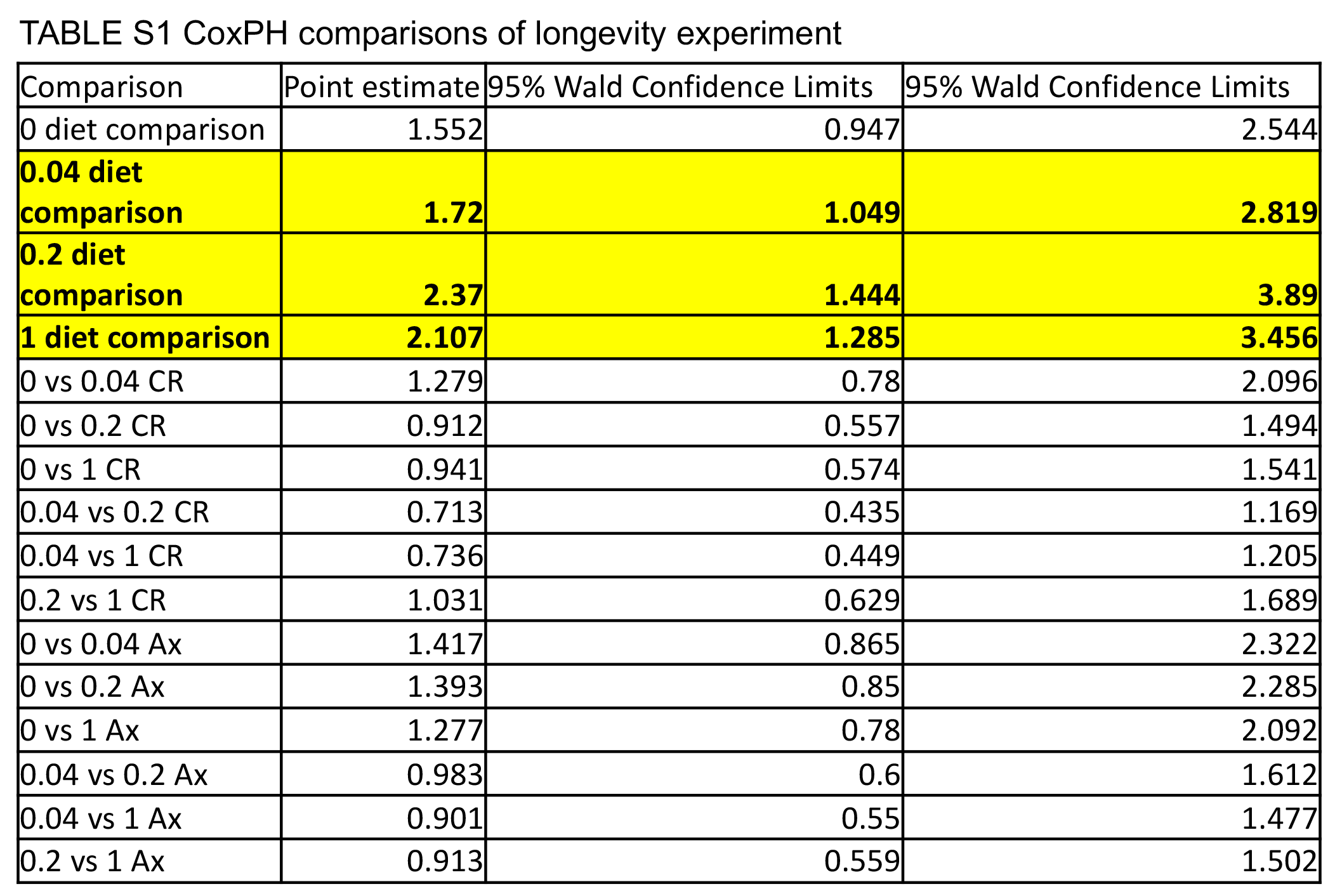

Supplement: TABLE S1 [file mbo001183761st1.tif]

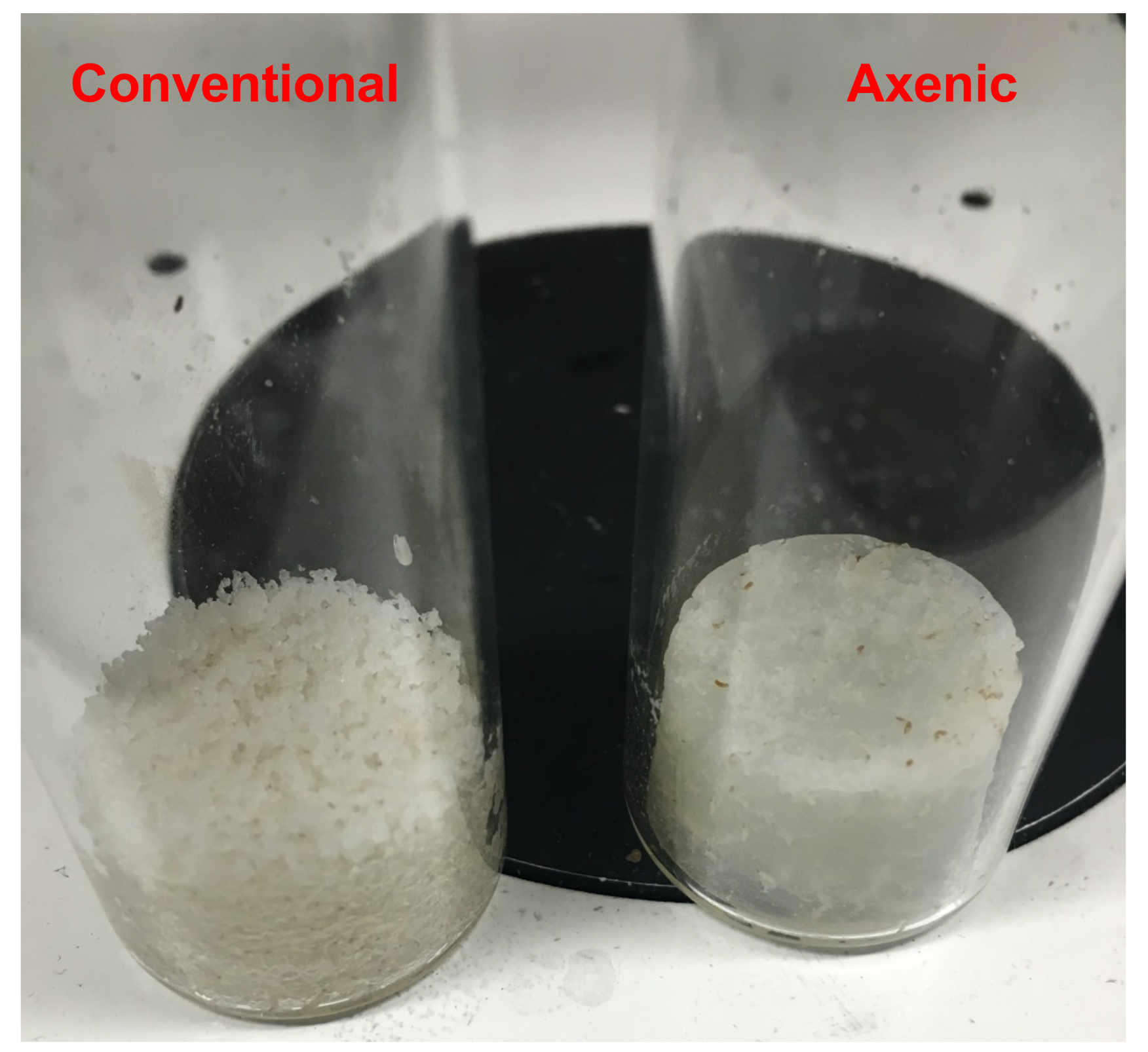

Supplement: FIG S1 [file mbo001183761sf1.tif]

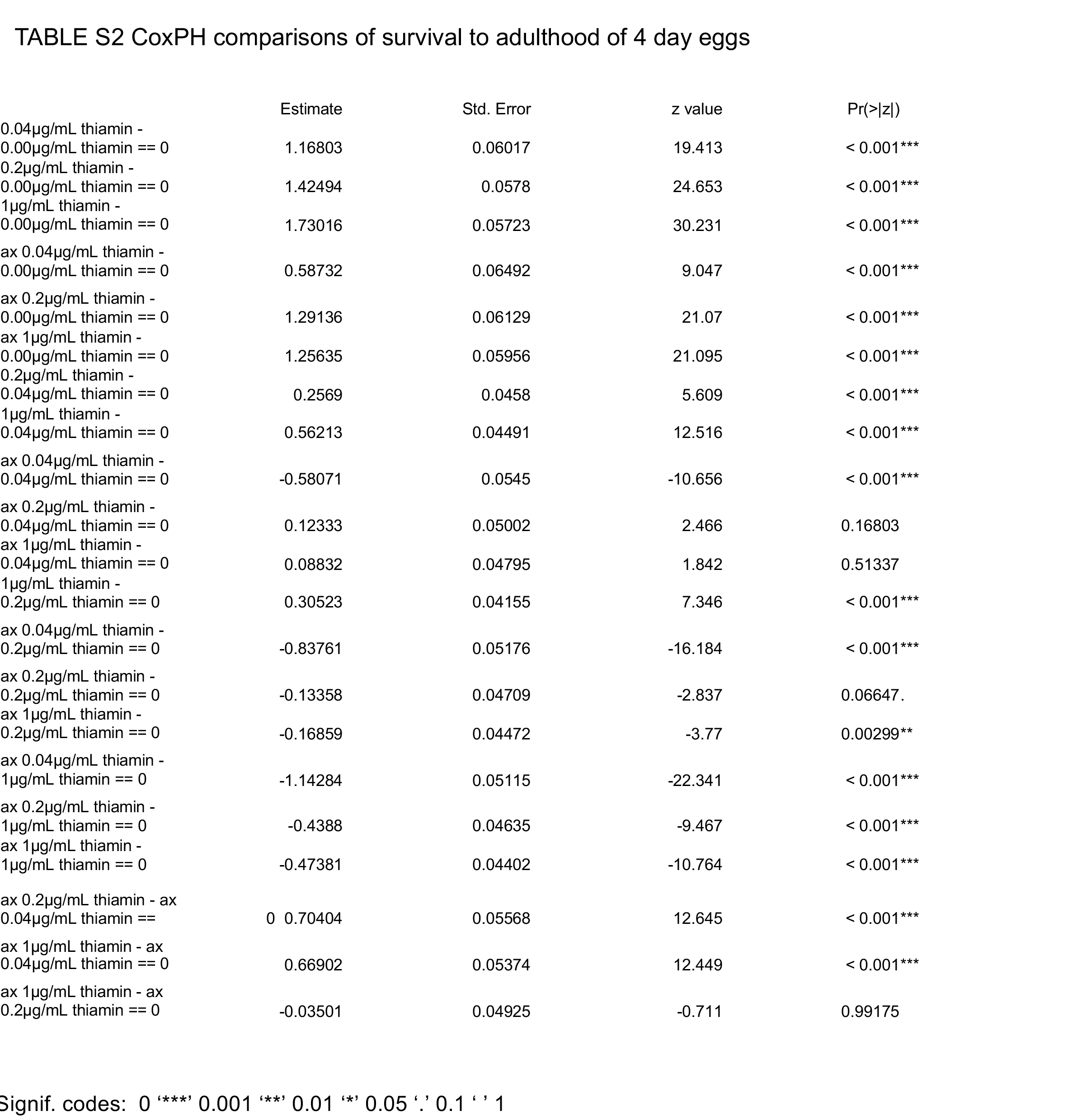

Supplement: TABLE S2 [file mbo001183761st2.tif]

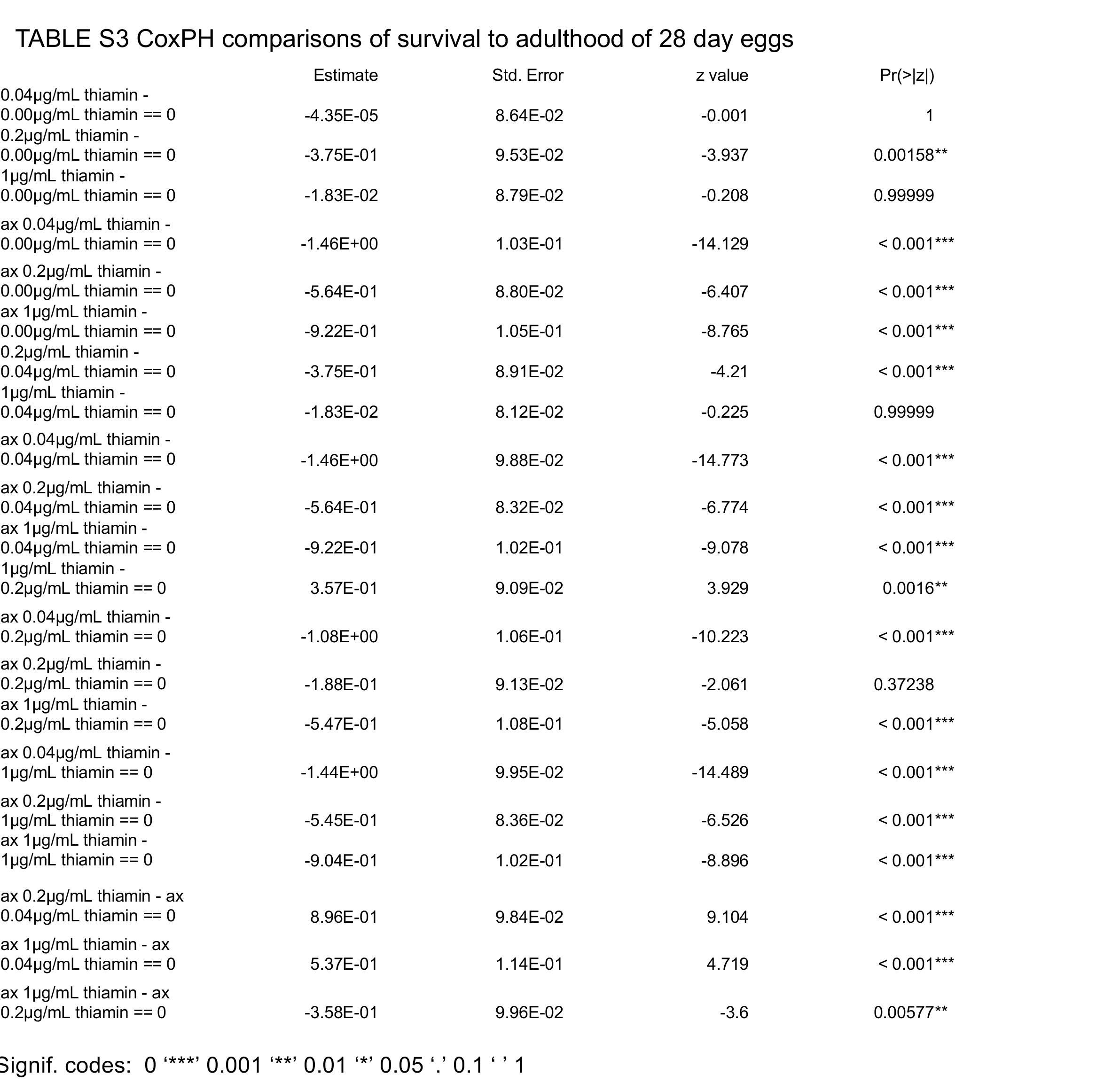

Supplement: TABLE S3 [file mbo001183761st3.tif]

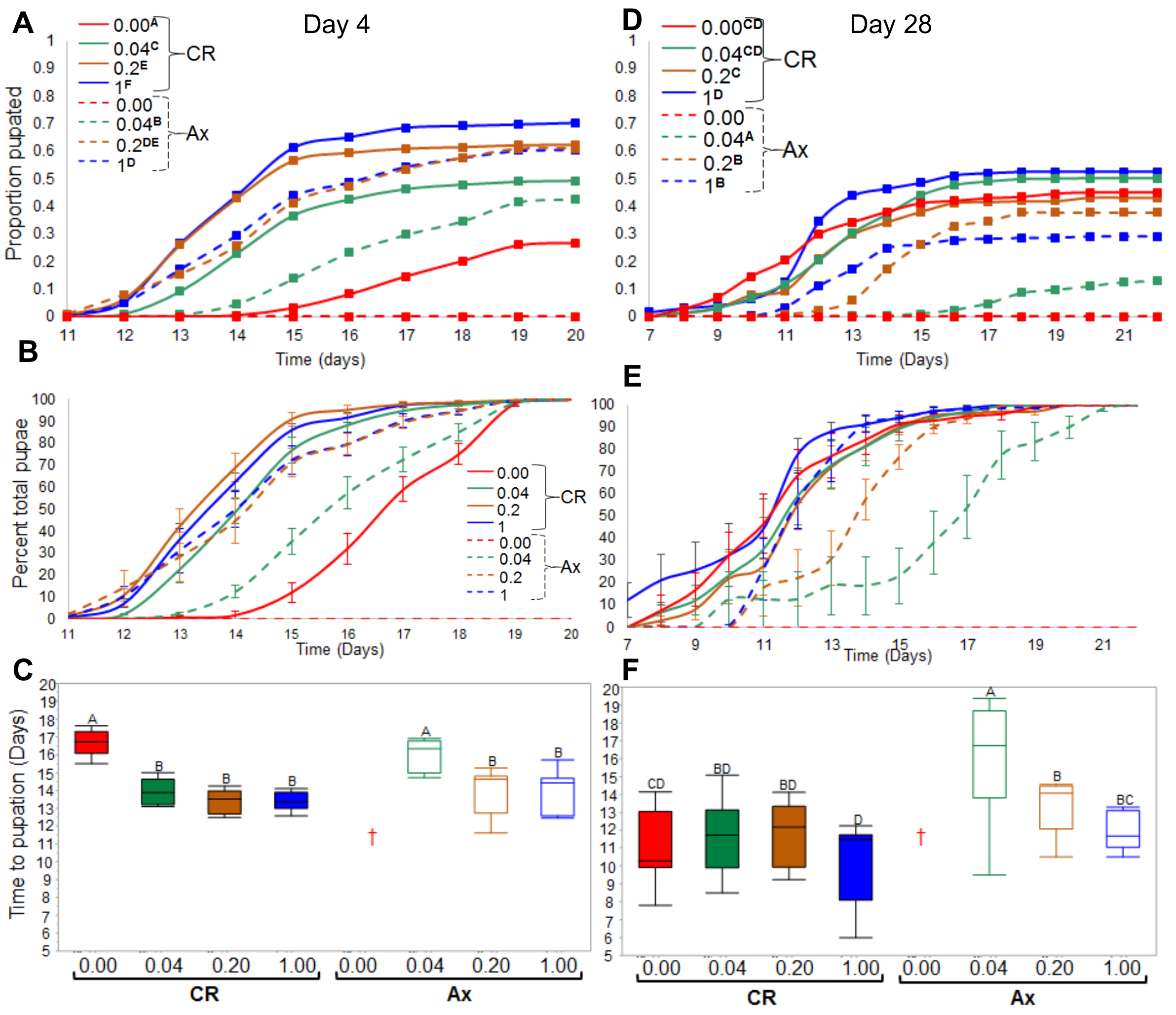

Supplement: FIG S2 [file mbo001183761sf2.tif]

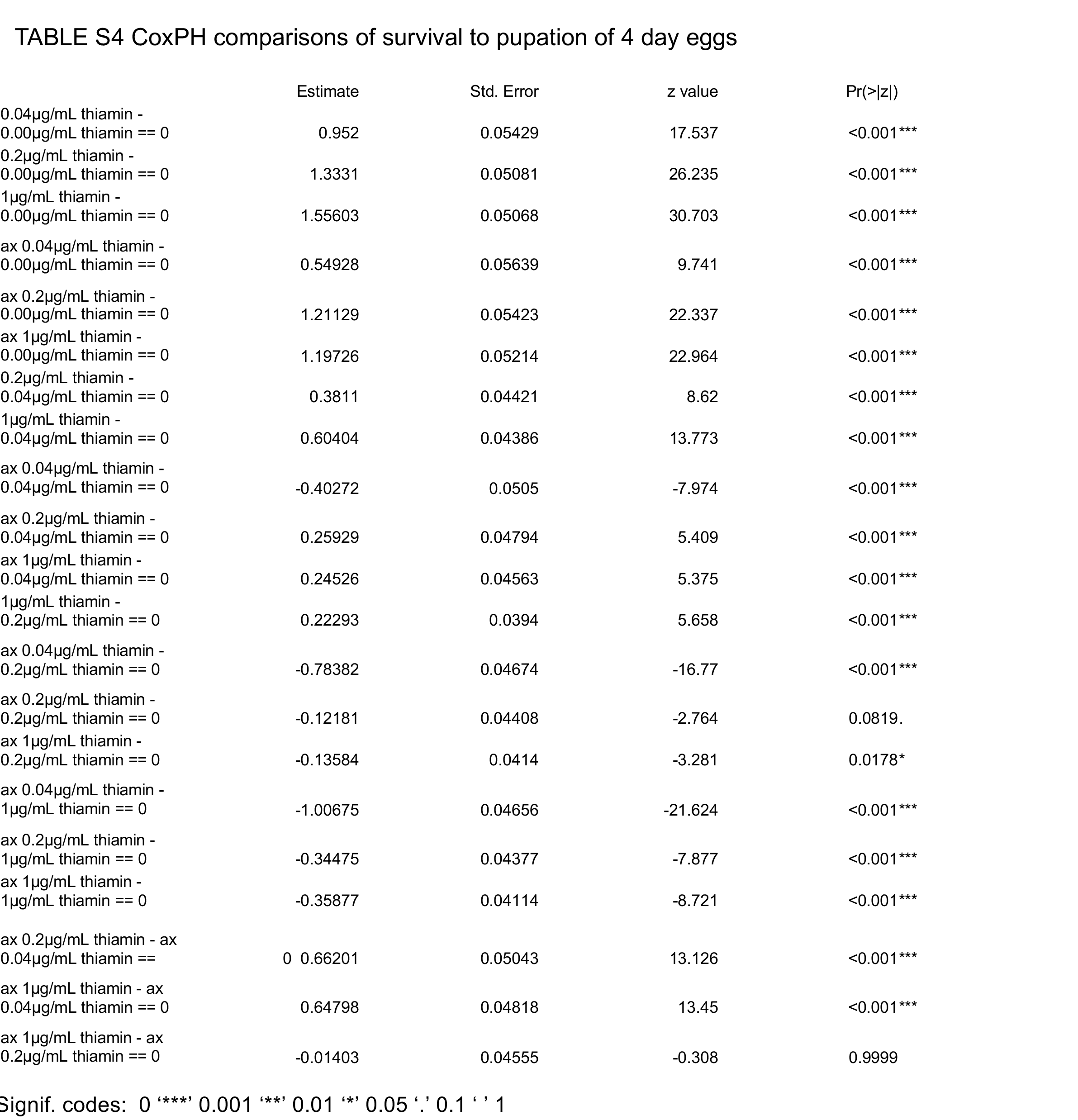

Supplement: TABLE S4 [file mbo001183761st4.tif]

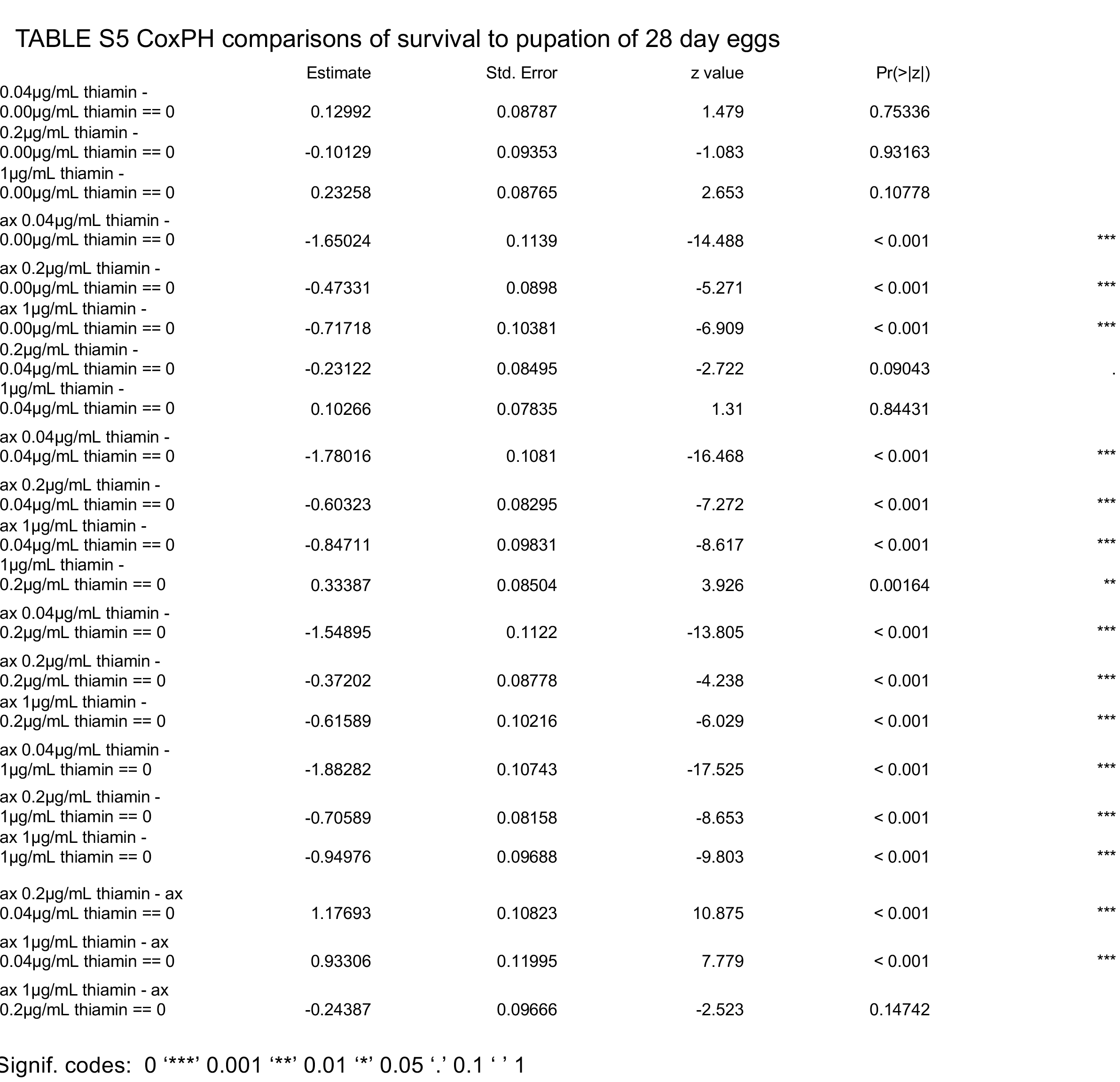

Supplement: TABLE S5 [file mbo001183761st5.tif]

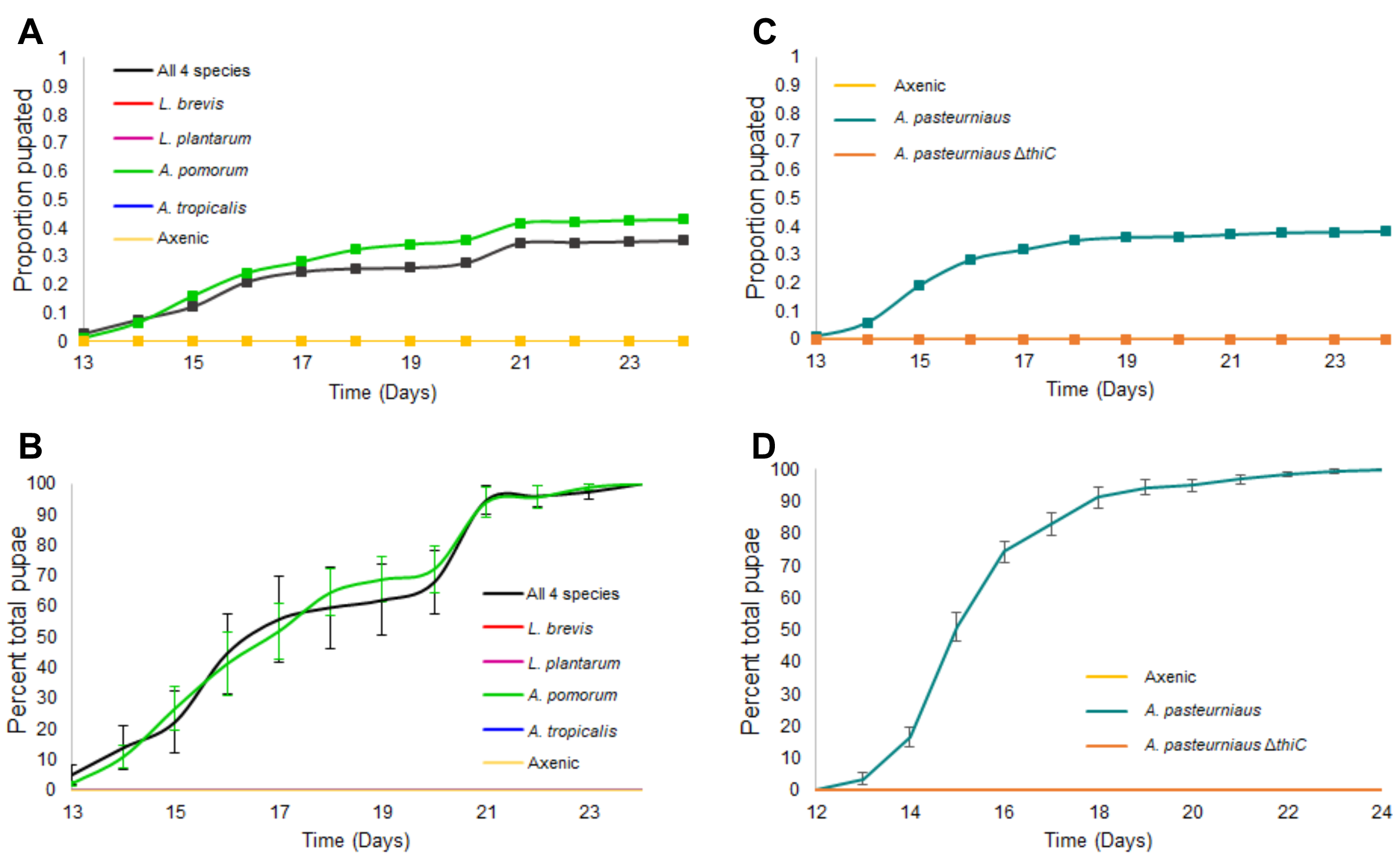

Supplement: FIG S3 [file mbo001183761sf3.tif]

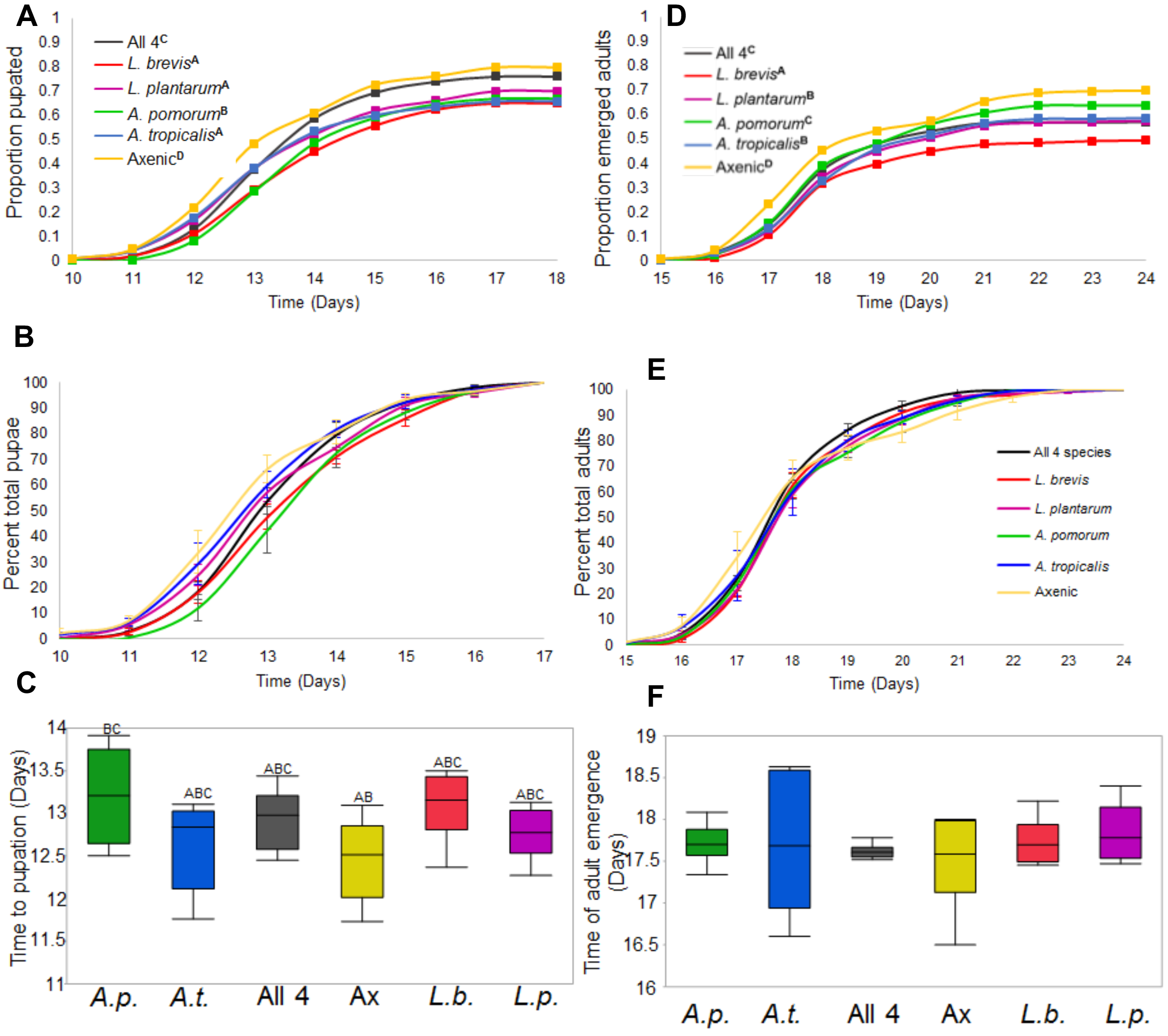

Supplement: FIG S4 [file mbo001183761sf4.tif]

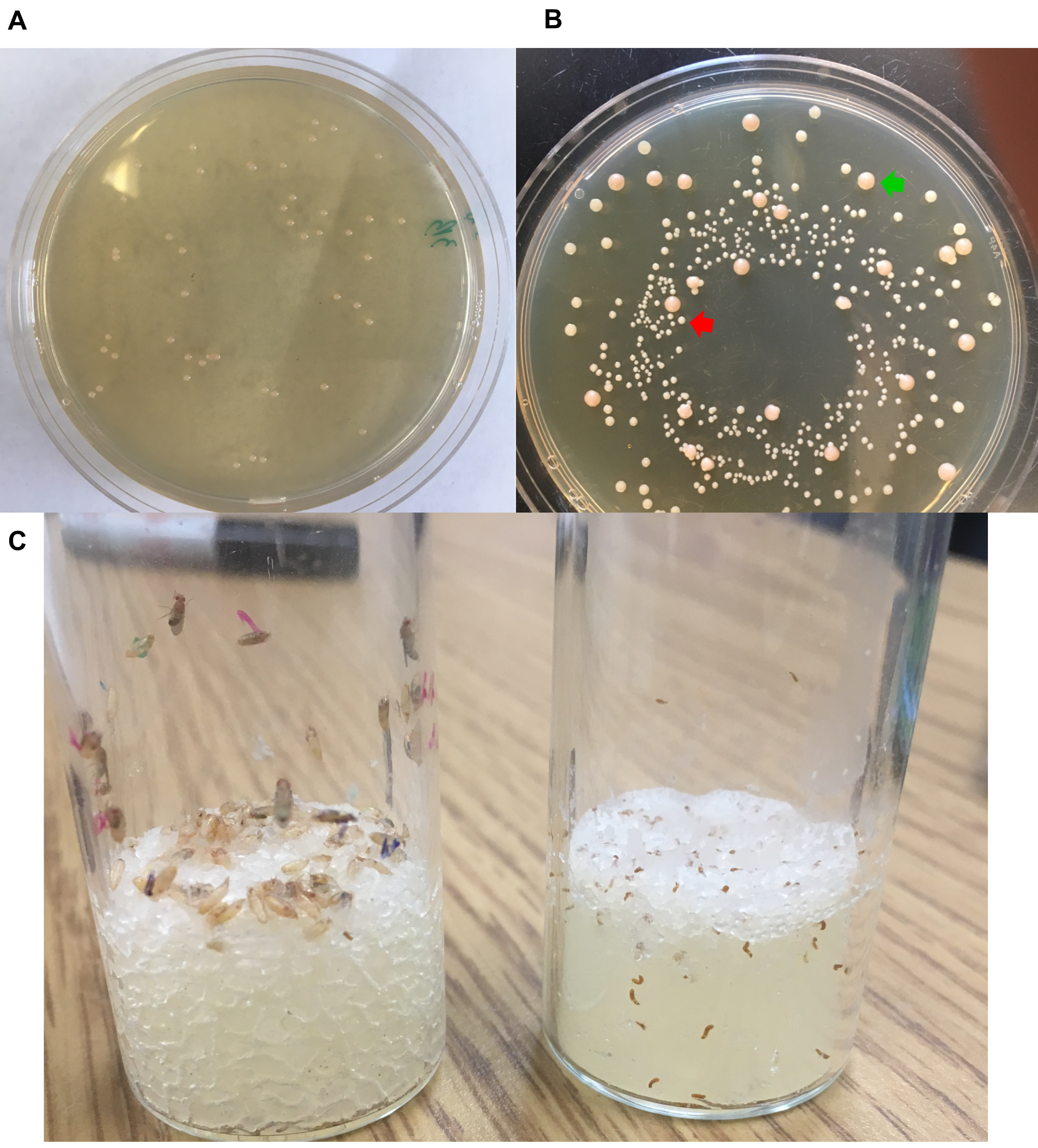

Supplement: FIG S5 [file mbo001183761sf5.tif]
